# Global evolution dynamics of genotype VI NDVs and dissection of the biological properties of strains from the prevalent sub-genotypes

**DOI:** 10.1128/jvi.01799-25

**Published:** 2025-12-30

**Authors:** Tao Di, Zongxi Han, Huixin Li, Yuhao Shao, Junfeng Sun, Shengwang Liu

**Affiliations:** 1 Division of Avian Infectious Diseases, State Key Laboratory of Animal Disease Control and Prevention, Harbin Veterinary Research Institute, the Chinese Academy of Agricultural Sciences 111613 , Harbin, the People’s Republic of China; Emory University School of Medicine, Atlanta, Georgia, USA

**Keywords:** Newcastle disease virus, sub-genotype VI.2.1.1.2.2, genetic evolution, replication, virulence

## Abstract

As a unique antigenic variant of Newcastle disease virus (NDV) in pigeons, genotype VI NDVs cause serious disease in pigeons and pose a potential threat to domestic poultry. In this study, analyses of genetic variation and evolution of global genotype VI NDVs revealed the worldwide distribution and continuous evolution of genotype VI NDVs. Of importance, VI.2.1.1.2.1 and VI.2.1.1.2.2 are the two prevalent sub-genotypes worldwide. The virulence, replication capacity, and pathogenesis in chickens of representative strains from VI.2.1.1.2.1 and different clusters in VI.2.1.1.2.2 were further evaluated. Compared with the VI.2.1.1.2.1 strain, increased replication capacity and virulence in chickens were observed in strains of VI.2.1.1.2.2. VI.2.1.1.2.2 strains showed preferential binding for α-2,3-linked sialic acids and superior performance in viral entry, cell-cell fusion, and release of progeny virions. Evaluation with recombinant viruses demonstrated that residues at positions 365 and 497 in the HN protein contributed to the differences in biological characteristics of VI.2.1.1.2.2 strains, and residue 365 was a key determinant. Moreover, our results showed that the activity of the viral replication complex contributed to the differences in replication capacity among these viruses, with the P protein being the major individual contributor. The optimal effect was achieved when the NP and L proteins were homologous. Moreover, the 5′ terminal trailer region was also found to be involved in the replication capacity of genotype VI NDVs; however, the viral V protein was not related to the replication and virulence of these viruses. Our findings highlight the potential risk of VI.2.1.1.2.2 NDVs due to their persistent circulation and evolution.

**IMPORTANCE:**

Genotype VI is the most diverse group of Newcastle disease viruses (NDVs). In addition to infectious disease in pigeons, the potential threat to chicken flocks and the public health implications associated with genotype VI NDVs also need to be addressed. Herein, comprehensive genetic evolution analysis revealed a global distribution pattern and continuous evolution of genotype VI NDVs worldwide. The biological characteristics of genotype VI NDVs belonging to different sub-genotypes were also evaluated. In particular, the widespread transmission, circulation, and constant evolution of currently prevalent sub-genotype VI.2.1.1.2.2 have led to the alteration of receptor binding preference, an increase in replication capacity, and a resultant increase in virulence in chickens. These findings expand our current understanding of the evolution and pathogenesis of genotype VI NDVs.

## INTRODUCTION

Newcastle disease virus (NDV), recently designated as *Avian orthoavulavirus 1*, is a member of the genus *Orthoavulavirus*, family *Paramyxoviridae* (1). As a ubiquitous poultry pathogen, NDV can infect more than 200 bird species, including domestic and wild birds worldwide (2). Due to widespread circulation in multiple bird species, NDV displays significant genetic diversity. All identified NDV strains belong to a single serotype but can be separated into two genetic groups, class I and class II, containing 1 and 20 defined genotypes, respectively (3).

Pigeon paramyxovirus type 1 (PPMV-1) is considered a unique antigenic variant of NDV, originating from the host adaptation mechanism of older virulent NDV in Columbiform birds (4, 5). The majority of PPMV-1 isolates belong to class II genotype VI and are responsible for the Newcastle disease (ND) outbreaks in pigeon species (6, 7). Viruses of genotype VI have been endemic in worldwide pigeon populations since their first emergence in the Middle East in the 1970s (8); these viruses show high genetic diversity and are currently divided into seven sub-genotypes (VI.1, VI.2.2.1, VI.2.2.2, VI.2.1.2, VI.2.1.1.1, VI.2.1.1.2.1, and VI.2.1.1.2.2) (3, 6, 9).

In China, PPMV-1 was first identified in 1985 from exotic pigeons and then spread widely in pigeon populations over time (10). Earlier China PPMV-1 isolates belonged to sub-genotype VI.1 (former VIb). Thereafter, sub-genotypes VI.2.2.2 (former VIe), VI.2.1.1.2.1 (former VIj), and VI.2.1.1.2.2 (former VIk) were identified (11–14). Recent epidemiology surveillance showed that the worldwide dispersal and evolution of genotype VI NDVs are ongoing (6, 13, 15). However, a comprehensive investigation of the distribution, genetic diversity, and evolution of genotype VI NDVs at a worldwide level has not been reported.

Genotype VI NDVs generally possess multiple basic amino acids at the fusion (F) protein cleavage site, which is a molecular characteristic of virulent NDV. This is consistent with the pathogenic phenotype of genotype VI NDVs in pigeons (16). However, determination of virulence via the standard intracerebral pathogenicity index (ICPI) in 1-day-old chicks indicates genotype VI NDVs are low or intermediate virulence for chickens. Most genotype VI strains cause minimal or mild clinical disease in chickens; however, these viruses could achieve high virulence after serial passages in chickens (17, 18). Though genotype VI NDVs are highly adapted to pigeons, the capacity of viruses in this genotype for interspecies transmission has been revealed (19–21). Historically, sporadic spread of genotype VI NDVs from pigeons to chickens and consequent ND outbreaks in chicken flocks have occurred (22, 23). A recent outbreak of ND in laying hens due to infection of sub-genotype VI.2.1.1.2.2 NDV from wild bird origin was reported in Switzerland in 2022 (24). The continuous circulation of genotype VI NDVs in wild birds and pigeons and inevitable contact of these birds with domestic chickens indicate that the risk of genotype VI NDVs to transmission to domestic poultry and resulting clinical disease should be taken into account. Notably, different viral sub-genotypes of genotype VI may exhibit distinct replicative or pathogenic properties in chickens, even though these viruses contain identical virulent F protein cleavage motifs (13, 25, 26). However, our understanding of the pathogenicity characteristics of genotype VI NDVs that belong to different sub-genotypes in chickens, especially the factors involved in virulence and replication capacity of these viruses, is still lacking. Furthermore, although NDV primarily affects birds, this virus can also infect non-avian species, including humans (27–32). The most important aspect is the fatal human infections that have been reported in France, which were associated with the VI.2.1.1.2.2 strain of NDV (personal communication from the 21st Annual Meeting of the National Reference Laboratories for AI and ND). This highlights the possible zoonotic features of the VI.2.1.1.2.2 NDV and emphasizes the importance of surveillance of the virus.

In this study, a systematic phylogenetic analysis of global genotype VI NDVs was performed. The virulence, replication capacity, and pathogenesis in chickens of the representative strains from sub-genotypes VI.2.1.1.2.1 and VI.2.1.1.2.2, the two dominant sub-genotypes prevalent worldwide, were also evaluated. Comparative analyses of biological properties among these viruses were performed, focusing on receptor-binding preference, entry efficiency, fusion activity, viral release efficiency, contribution of viral factors to virus replication, and interferon (IFN) antagonism. Our results indicate that alteration of receptor binding preference and increase of entry and replication capacities directly contribute to the increased virulence of sub-genotype VI.2.1.1.2.2 NDV in chickens.

## RESULTS

### Evolution dynamics and distribution of genotype VI NDVs worldwide

**Fig 1:**
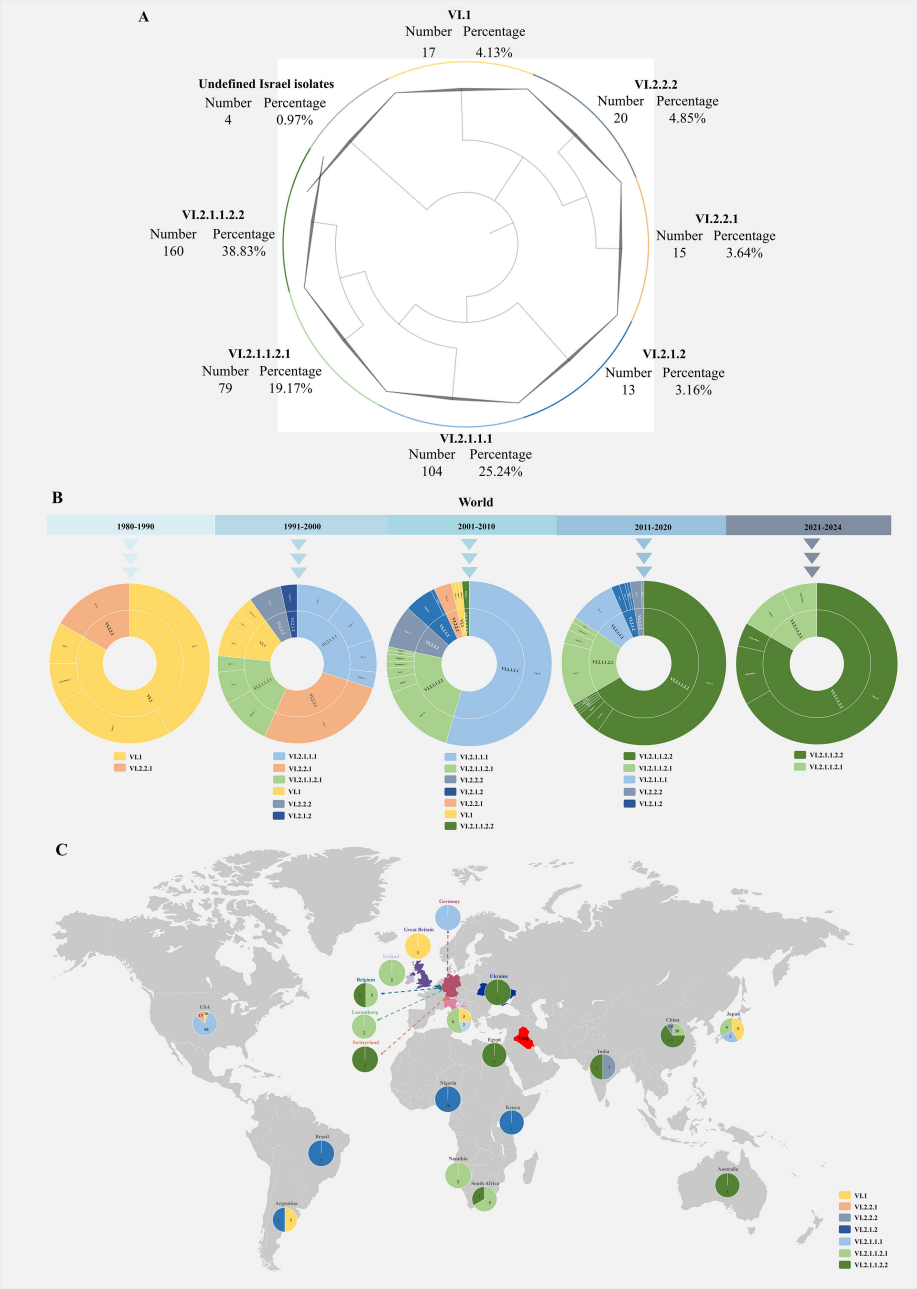
Phylogenetic analysis of genotype VI NDVs. (**A**) The F gene CDSs of 412 strains of genotype VI NDV were used for phylogenetic analysis. Phylogenetic trees were constructed with MEGA 10 software using maximum likelihood with 1,000 bootstraps. The complete phylogenetic tree is shown in Fig. S1. The number and percentage of strains in each sub-genotype are shown. (**B**) Temporal distribution of genotype VI NDVs worldwide. Numbers of strains and the corresponding countries in each sub-genotype during the indicated time periods are shown. (**C**) Geographic distribution of genotype VI NDVs worldwide. The sub-genotype and numbers of strains identified in the indicated countries are shown. The map was sourced from the Database of Global Administrative Areas (GADM) 3.6.

The phylogenetic analysis using all available coding sequences (CDSs) of F genes from genotype VI NDVs in GenBank (*n* = 412) showed that, except for four unclassified Israel isolates, all genotype VI isolates were grouped into sub-genotype VI.1 (*n* = 17), VI.2.2.2 (*n* = 20), VI.2.2.1 (*n* = 15), VI.2.1.2 (*n* = 13), VI.2.1.1.1 (*n* = 104), VI.2.1.1.2.1 (*n* = 79), and VI.2.1.1.2.2 (*n* = 160) (Fig. 1A; Fig. S1 and Table 1).

**TABLE 1:** Unique nucleotide substitutions in F CDS of sub-genotype VI.2.1.1.2.2 NDVs isolated from different countries except China^*a*^

Description	Substitution and position	No.
Unique substitutions in Belgium isolates	C99T, C819T, T907C, and C999T	4
Substitutions shared by Australia and South Africa isolates	C14T, C81T, C276T, C978A, T984C, A1296T, T1536G, and C1629T	8
Unique substitutions in Australia isolates	C510T, T621C, T757C, T1179C, and A1263G	5
Unique substitutions in South Africa isolates	A17G, C252T, C262T, A370G, T378C, A549G, T654C, C688T, C870T, A881G, C900T, T921C, T1266C, A1281G, T1341C, C1462T, C1467T, C1473T, C1524T, G1558A, C1569T, and C1649A	22
Unique substitutions in Switzerland isolates	A11G, G24A, C28T, C29T, C59T, A147G, G187A, C224T, A291C, C294T, T741C, C801T, A815G, G963A, C1005A, C1149T, C1300T, A1374T, C1395T, A1453G, A1470G, A1521G, and T1555C	23

^*a*^ Only one VI.2.1.1.2.2 isolate was identified from Egypt, India, and Ukraine, respectively. Thus, the nucleotide substitutions in these three isolates were not marked.

As shown in Fig. 1B, the early strains identified during 1980–1990 were assigned to sub-genotypes VI.1 and VI.2.2.1. During 1991–2000, strains belonging to sub-genotypes VI.2.1.1.1, VI.2.1.1.2.1, VI.2.2.2, and VI.2.1.2 emerged. In the first decade of the 21st century, in addition to previously reported sub-genotypes, isolates from Belgium assigned to sub-genotype VI.2.1.1.2.2 were first identified. In the following decade, the early sub-genotypes VI.1 and VI.2.2.1 were no longer reported, whereas viruses belonging to the other five sub-genotypes were continuously identified. Notably, VI.2.1.1.2.2 and VI.2.1.1.2.1 became the two predominant sub-genotypes during this period. Thereafter, all reported genotype VI isolates were assigned to these two sub-genotypes.

Further analysis showed that genotype VI NDVs exhibit a global distribution pattern and have been isolated in all continents except Antarctica (Fig. 1C). Briefly, European isolates from Germany, Great Britain, Ireland, Belgium, Luxembourg, Switzerland, Italy, and Ukraine were assigned to sub-genotypes VI.1, VI.2.1.1.1, VI.2.1.1.2.1, and VI.2.1.1.2.2. Asian isolates from China, Japan, and India were assigned to sub-genotypes VI.1, VI.2.2.2, VI.2.1.1.1, VI.2.1.1.2.1, and VI.2.1.1.2.2. African isolates from Egypt, Nigeria, Kenya, Namibia, and South Africa were assigned to sub-genotypes VI.2.1.2, VI.2.1.1.2.1, and VI.2.1.1.2.2. In Oceania, sub-genotype VI.2.1.1.2.2 was identified in Australia. In North America, sub-genotypes VI.1, VI.2.1.1.1, VI.2.2.1, and VI.2.1.1.2.1 were identified in the United States. In South America, isolates from Brazil and Argentina were assigned to sub-genotypes VI.1 and VI.2.1.2. Collectively, these findings demonstrate the evolutionary dynamics and worldwide distribution of genotype VI NDVs.

### Prevalence of genotype VI NDVs in China

**Fig 2:**
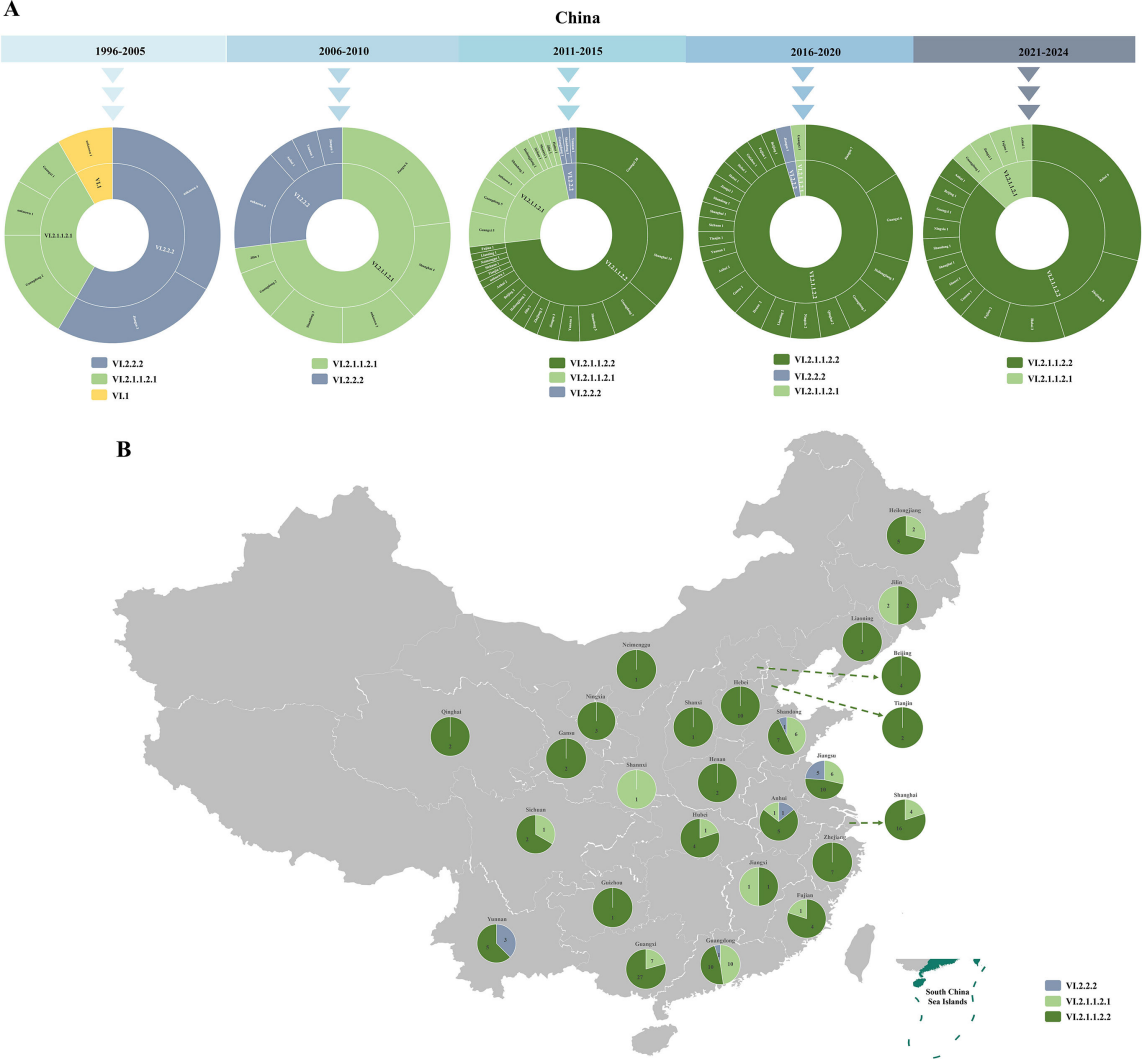
Temporal and geographic distribution of genotype VI NDVs in China. (**A**) Temporal distribution of genotype VI NDVs. Numbers of strains and the corresponding locations for each sub-genotype during the indicated time periods are shown. (**B**) Geographic distribution of genotype VI NDVs. The sub-genotype and numbers of strains identified in the indicated locations are shown. The map was sourced from the Database of Global Administrative Areas (GADM) 3.6.

In China, isolates identified during 1996–2005 were assigned to sub-genotypes VI.1, VI.2.2.2, and VI.2.1.1.2.1. Since then, strains belonging to the oldest sub-genotype VI.1 were not recorded. Sub-genotypes VI.2.2.2 and VI.2.1.1.2.1 were continuously identified between 2006 and 2020, but the number of isolated strains gradually decreased over time. There has been no report of isolation of sub-genotype VI.2.2.2 since 2021. Sub-genotype VI.2.1.1.2.2 strains were first described in 2011 in six provinces in China. Since then, the number of strains of this sub-genotype has gradually increased, although there are still sporadic isolations of VI.2.1.1.2.1 strains (Fig. 2A). The geographic distribution of 190 Chinese genotype VI strains with available location information showed that 11 sub-genotype VI.2.2.2 strains were identified from five provinces, 43 sub-genotype VI.2.1.1.2.1 strains from 13 provinces, and 136 sub-genotype VI.2.1.1.2.2 strains from 25 provinces. These data demonstrate that four sub-genotypes have appeared in China. Among them, VI.1 and VI.2.2.2 have not spread widely over time, whereas VI.2.1.1.2.1 and VI.2.1.1.2.2 show nationwide distributions, in which VI.2.1.1.2.2 has become the predominant sub-genotype since 2011 (Fig. 2B).

### Molecular characteristics of the F gene of sub-genotype VI.2.1.1.2.2 NDVs

Phylogenetic analysis indicated that all VI.2.1.1.2.2 NDVs could be traced back to the first VI.2.1.1.2.2 strain, PPMV-1/Belgium/05-03936-8/2005 (Belgium/05), which was isolated from a pigeon in 2005 in Belgium (33). Sequence analysis of the F gene CDS among isolates from Belgium and other countries, except China, showed that four unique substitutions were found in three Belgian strains isolated in 2011. In addition, 5, 22, and 23 unique substitutions were observed in isolates from Australia, South Africa, and Switzerland, respectively (Table 1 and Fig. S2). These findings suggest that independent evolution of VI.2.1.1.2.2 NDVs has occurred in these countries.

Virus strains of sub-genotype VI.2.1.1.2.2 isolated in China presented some specific nucleotide mutations and were divided into six clusters based on the nucleotide substitution patterns; each cluster comprised at least five strains showing identical nucleotide substitution patterns. Cluster 1 included strains that were obtained from 10 provinces and were closely related to Belgium/05 and shared six unique nucleotides with it, distinguishing them from strains in the other five clusters. Chinese strains in cluster 1 also shared four additional unique substitutions. Cluster 2 contained strains from three provinces and showed 18 unique substitutions. Meanwhile, 3, 1, and 10 unique substitutions were found in clusters 3, 4, and 5, which were composed exclusively of strains from Guangxi, Shanghai, and Guangdong, respectively. Moreover, 40 strains from 17 provinces were grouped into cluster 6, the largest cluster, which possessed six unique substitutions (Table 2 and Fig. S3).

**TABLE 2:** Unique nucleotide substitutions in F CDS among China sub-genotype VI.2.1.1.2.2 NDVs

Description	Nucleotide and position	No.
Unique nucleotides shared by Belgium/05 and China isolates in cluster 1	G5, T38, G411, A585, A699, and G1254	6
Unique substitutions commonly shared by clusters 2-6	G5A, T38C, G411A, A585G, A699G, and G1254T	6
Unique substitutions shared by China isolates in cluster 1	G201A, T1033C, C1455T, and A1596G	4
Unique substitutions in cluster 2	A219G, T270C, C279T, T315C, G396A, G859A, T1080C, T1083C, T1095C, C1100T, A1146G, C1156T, C1176T, C1272T, A1419G, A1469G, C1524T, and T1650C	18
Unique substitutions in cluster 3	C1131G, T1203C, and G1227A	3
Unique substitutions in cluster 4	G53A	1
Unique substitutions in cluster 5	A243G, G246A, C507T, G519A, A966G, G1065T, A1079C, A1274G, A1427G, and G1457A	10
Unique substitutions in cluster 6	G74A, T168C, A1263G, C1431T, C1542T, and A1589G	6

Only a few consistent amino acid substitutions were found in each cluster. Compared with cluster 1, G2D and L13P substitutions were found in the signal peptide of F protein in the other five clusters. V287I, T367I, L386F, and K490R substitutions were found in cluster 2. No consistent substitution was found in cluster 3. An R18Q substitution was found in cluster 4, and four substitutions (M355I, Y360S, N476S, and S486N) were identified in cluster 5. A unique C25Y substitution in the signal peptide and a K530R substitution in the cytoplasmic tail were found in cluster 6 (Fig. S4). It is noteworthy that strains in clusters 1, 3, 4, and 5 were mainly obtained between 2011 and 2015, while almost all strains isolated since 2015 were grouped into clusters 2 (*n* = 5) and 6 (*n* = 40). Taken together, these data suggest that Chinese VI.2.1.1.2.2 NDVs might originate from an ancestor from Belgium, and divergent and progressive evolution has occurred over time.

### Determination of virulence and replication capacity

Representative strains, including pi/CH/LHLJ/110813 (P0813) in VI.2.1.1.2.1 and pi/CH/LLN/110713 (P0713) and pi/CH/LHLJ/170506 (P0506) in VI.2.1.1.2.2 (34, 35) were selected for further research. P0713, the first identified sub-genotype VI.2.1.1.2.2 strain in China, belongs to cluster 1 and is closely related to the earliest Belgium/05 strain. Strain P0506 belongs to cluster 6, which possessed unique substitutions (Fig. S1 and S3). To rule out the potential confounding effects from possible viral subpopulations, recombinant viruses designated rP0813, rP0713, and rP0506 were recovered via *de novo* reverse genetic systems. Subsequently, the mean death time (MDT) and ICPI assays were performed to evaluate viral virulence. The MDTs of P0813, P0713, and P0506 were 110 h, 88 h, and 58 h, and the ICPIs were 1.18, 1.23, and 1.45, respectively. Comparably, the MDTs of rP0813, rP0713, and rP0506 were 120 h, 76 h, and 55 h, and the ICPIs were 1.18, 1.28, and 1.41, respectively. These results indicate that P0813, P0713, and P0506 exhibited a sequentially increasing trend in virulence, and the recovered recombinant viruses were equivalent in virulence to their biological parents.

**Fig 3:**
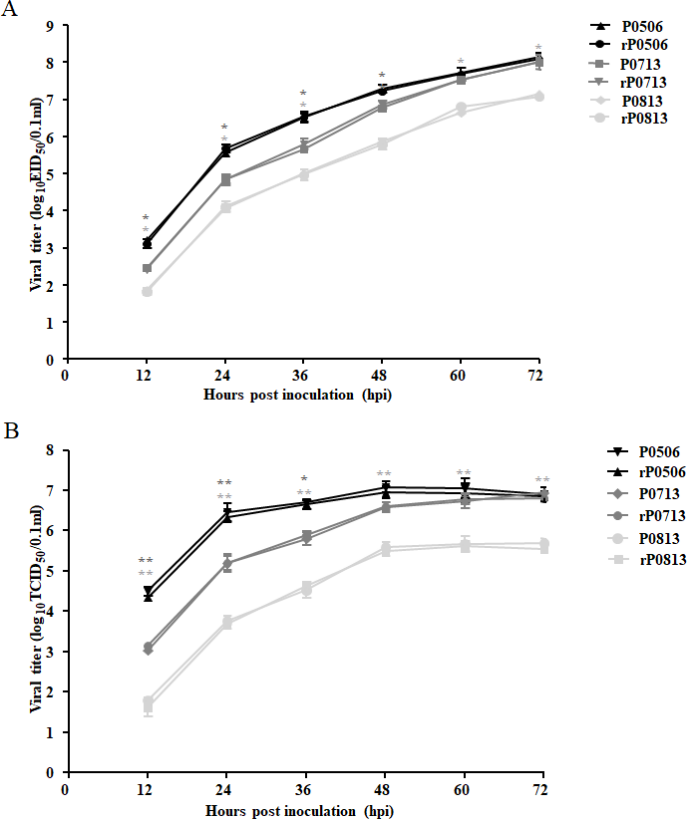
The replication capacity of VI.2.1.1.2.1 and VI.2.1.1.2.2 strains *in ovo* and *in vitro*. (**A**) The growth kinetics of parent (P0813, P0713, and P0506) and recombinant (rP0813, rP0713, and rP0506) viruses *in ovo*. Each virus was inoculated into 9-day-old embryonated SPF eggs with 100 EID_50_. The allantoic fluids (AFs) from five eggs receiving each virus were harvested at the indicated time points, and viral titers were determined and expressed as EID_50_. (**B**) Growth kinetics of parent (P0813, P0713, and P0506) and recombinant (rP0813, rP0713, and rP0506) viruses *in vitro*. DF-1 cells were incubated with each of the viruses at a multiplicity of infection (MOI) of 0.01. Supernatants were collected at the indicated time points, and viral titers were determined and expressed as TCID_50_. Data are presented as means ± standard deviation (SD) and were analyzed using one-way analysis of variances (*P < 0.05; **P < 0.01; ***P < 0.001).

To compare the replication capacities of these viruses, the growth kinetics of both parental and recombinant viruses in SPF embryonated chicken eggs were first determined. As shown in Fig. 3A, the viral titers of P0713 and P0506 were significantly higher than those of P0813 at each time point. Comparatively, P0506 had a higher viral titer than P0713 at 12–36 h post-infection (hpi), while they showed comparable viral titers after 48 hpi. Subsequently, the replication capacities of these viruses were further determined in DF-1 cells. Similarly, the viral titers in P0713- and P0506-infected DF-1 cells were significantly higher than those of P0813-infected DF-1 cells at each time point. Strain P0506 showed higher viral titers than P0713 before 48 hpi (Fig. 3B). As expected, growth kinetics of each recombinant virus in eggs and DF-1 cells were indistinguishable from those of their respective parent viruses (Fig. 3A and B). These findings indicate that the replication capacities are likely directly related to the virulence phenotype of these viruses. For the construction of chimeric viruses, the recovered rP0813, rP0713, and rP0506 were used in subsequent studies.

### Evaluation of the pathogenic characteristics in chickens

**Fig 4:**
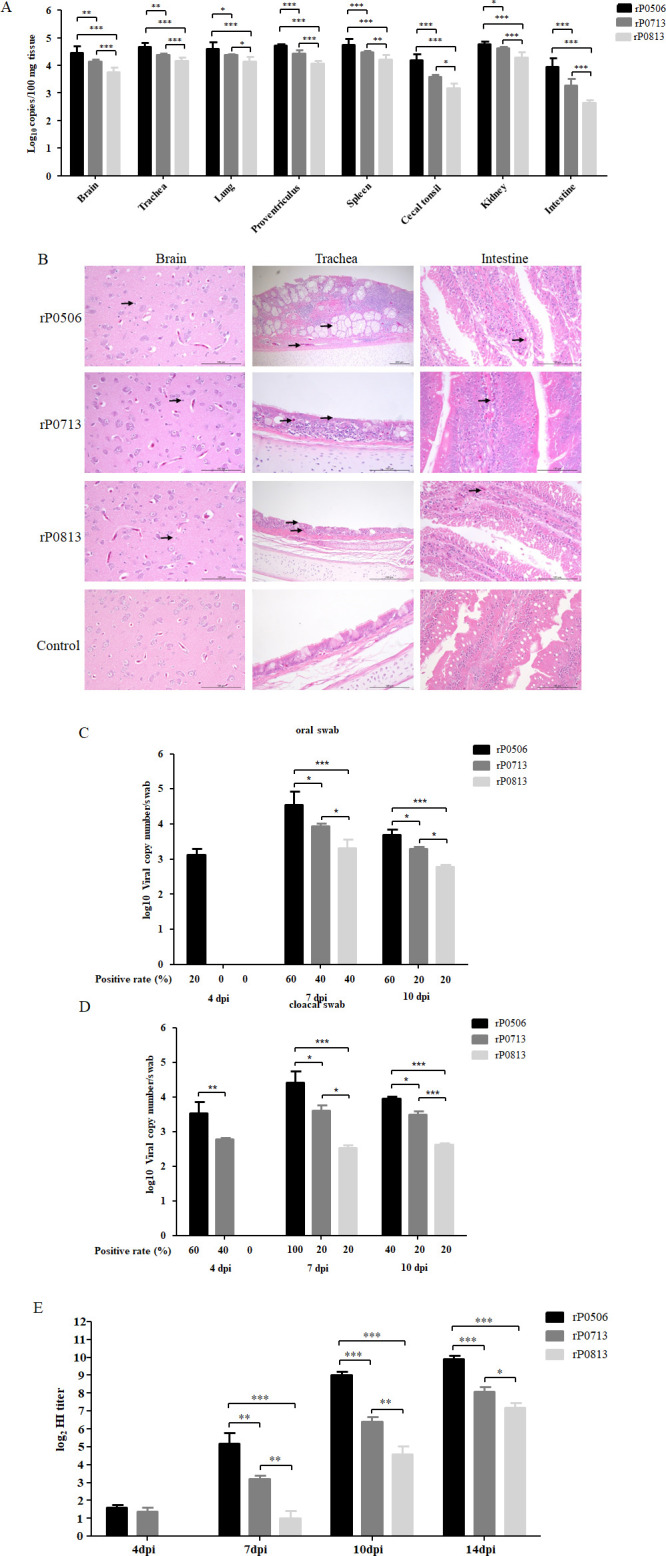
Pathogenicity evaluation of VI.2.1.1.2.1 and VI.2.1.1.2.2 strains in chickens. (**A**) Replication of rP0813, rP0713, and rP0506 *in vivo*. Four-week-old SPF chicks were infected with rP0813, rP0713, and rP0506 via the oculonasal route, with a dose of 10^6^ EID_50_/100 μL per chick. Five birds from each group were killed humanely at 4 dpi, and the tissue samples of the brain, trachea, lung, proventriculus, spleen, cecal tonsil, kidney, and intestine were collected. NDV viral RNA was extracted, and viral loads were quantified by real-time RT-PCR. (**B**) Histopathology of the brain, trachea, and intestine of chickens infected with rP0813, rP0713, and rP0506. Tissue samples from sacrificed chickens at 4 dpi were fixed with formalin, sectioned, and stained with hematoxylin and eosin. Lesions are indicated by black arrows. (**C and D**) Detection of virus shedding following infection with rP0813, rP0713, and rP0506. Oral (**C**) and cloacal (**D**) swabs were collected at 4, 7, 10, and 14 dpi. Viral loads were quantified by real-time RT-PCR. The average copy number of viral RNA in the positive samples is shown. Samples with a Ct value of <35 was considered NDV positive. (**E**) HI antibody titers in serum samples of chickens infected with rP0813, rP0713, and rP0506 were determined by HI assay. Data are presented as means ± SD and were analyzed using one-way analysis of variances (*P < 0.05; **P < 0.01; ***P < 0.001).

All chickens infected with rP0813 or rP0713 exhibited no apparent clinical signs and survived until the end of the animal experiments, similar to controls. One bird infected with rP0506 displayed mild depression at 6 days post-infection (dpi) and recovered on day 10. No gross lesions were observed in the tissues of the brain, trachea, lung, proventriculus, spleen, cecal tonsil, kidney, and intestine that were collected on 4 dpi from each group. As shown in Fig. 4A, viral RNA could be detected in all tissue samples from rP0813-, rP0713-, and rP0506-infected chickens. Notably, in each tissue sample, rP0813 showed the lowest level of viral load, and rP0506 presented a higher viral load than rP0713. No viral RNA was detected in tissues from the control group.

The brain, trachea, and intestine tissues were further subjected to histopathological examination; representative images for these tissues are shown (Fig. 4B). In the brain, rP0813, rP0713, and rP0506 caused mild diffuse proliferation of glial cells in the cortical region. In the trachea, lesions of local thickening of the tracheal mucosa, goblet cell hyperplasia, and inflammatory cell infiltration in the lamina propria and submucosa were observed in the rP0506 group. By contrast, the tracheal sections of the rP0813 and rP0713 groups exhibited mild inflammatory cell infiltration in the lamina propria and deciliation of tracheal epithelium. In the intestine, rP0506 caused mild inflammatory cell infiltration in the lamina propria of the intestinal mucosa and induced some cell necrosis. Similar lesions were also observed in the rP0813 and rP0713 groups. No pathological lesions were observed in chicken tissues from the control group.

Virus shedding through oral and cloacal routes was also examined. For oral swabs, the viral shedding rates in the rP0506 group at 4, 7, and 10 dpi were 20% (2/10), 60% (6/10), and 60% (6/10), respectively. In contrast, the rP0713 and rP0813 groups showed no shedding at 4 dpi, with shedding rates of 40% (4/10) and 20% (2/10) at both 7 and 10 dpi (Fig. 4C). For cloacal swabs, the shedding rates in the rP0506 group were 60% (6/10), 100% (10/10), and 40% (4/10) at 4, 7, and 10 dpi, respectively. The rP0713 group showed shedding rates of 40% (4/10), 20% (2/10), and 20% (2/10) at the same time points. The rP0813 group showed no shedding at 4 dpi but showed a 20% (2/10) shedding rate at both 7 and 10 dpi (Fig. 4D). No viral shedding was detected in any infected groups at 14 dpi, and all swabs from the control group were negative. Viral loads in both oral and cloacal swabs from the rP0506 group were significantly higher than those from the rP0713 and rP0813 groups at each time point, with the rP0713 group showing a higher viral load than the rP0813 group, which demonstrated the lowest viral load (Fig. 4C and D).

To evaluate the immune response induced by these viruses, hemagglutination inhibition (HI) antibodies in serum samples collected at 4, 7, 10, and 14 dpi were determined. As shown in Fig. 4E, HI antibodies were detectable from 7 dpi in all infected groups, and the antibody titers gradually increased with time. Comparatively, the HI antibody titers induced by rP0506 and rP0713 were significantly higher than those of rP0813 at each time point, and rP0506 induced a higher titer than rP0713.

Collectively, rP0506 demonstrated a higher-level pathogenicity in chickens, compared to that of rP0713 and rP0813. Notably, the replication capacity of these viruses appears to be responsible for their virulence and pathogenicity phenotype as previously reported (36, 37).

### Determination of receptor-binding property and cellular entry efficacy

**Fig 5:**
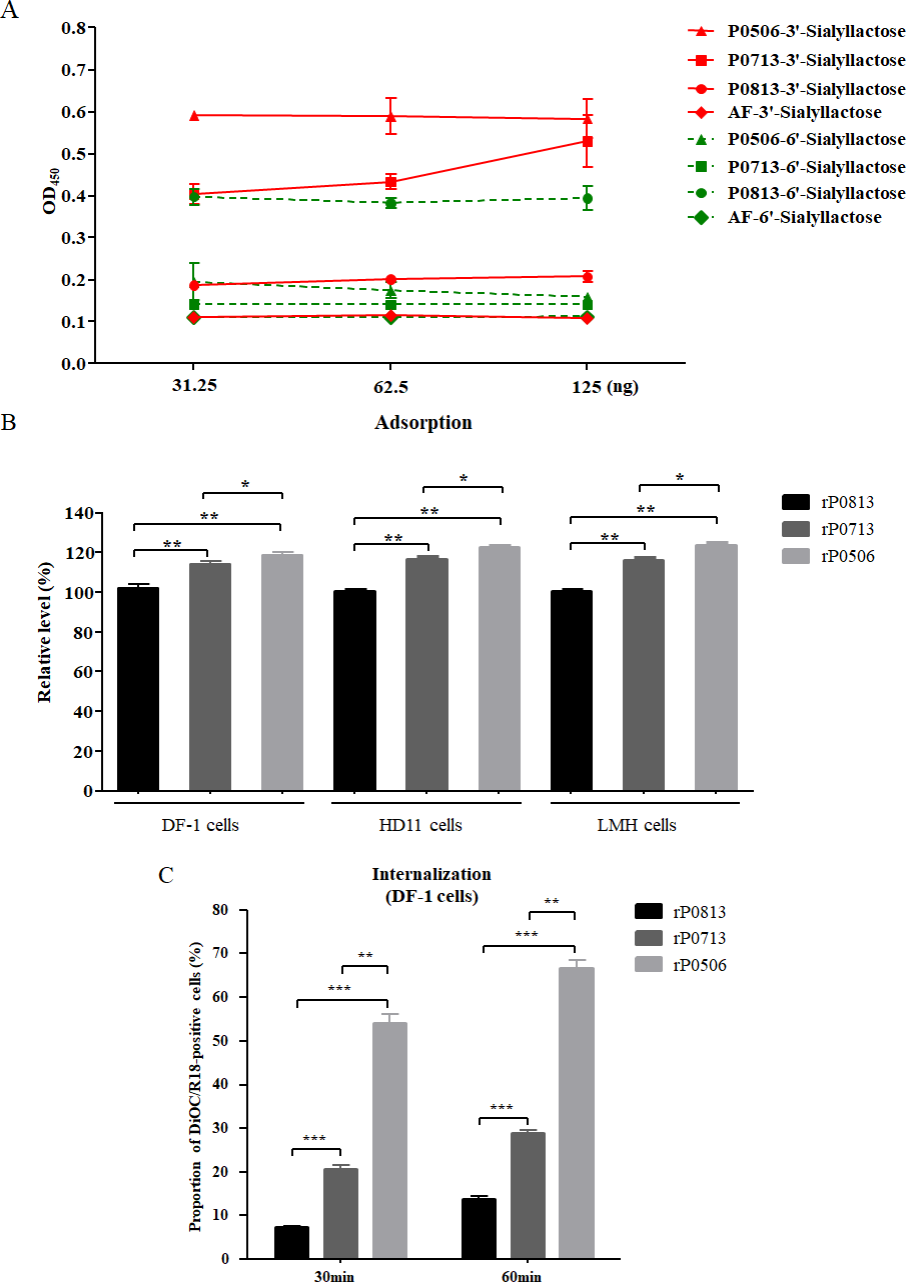
Evaluation of receptor binding and cellular entry efficacy. (**A**) Binding properties of rP0813, rP0713, and rP0506 to α-2,3-linked and α-2,6-linked sialic acid were evaluated via a solid-phase binding assay using 3′-sialyllactose-biotin and 6′-sialyllactose-biotin. (**B and C**) Cellular entry of rP0813, rP0713, and rP0506. (**B**) DF-1, HD11, and LMH cells were inoculated with rP0813, rP0713, and rP0506 for 1 h at 4°C to allow for viral adsorption. Viral RNA was quantified by real-time RT-PCR. Levels of viral RNA copies were calculated relative to those of rP0813-inoculated cells (×100%). (**C**) DiOC/R18 labeled rP0813, rP0713, and rP0506 were adsorbed onto DF-1 cells and incubated for 30 and 60 min at 37°C to permit internalization. Then the cells were washed, fixed, and subjected to flow cytometric analysis. The proportion of green- and red-double positive cells is shown. Data are presented as means ± SD and were analyzed using one-way analysis of variance (*P < 0.05; **P < 0.01; ***P < 0.001).

Initial infection of NDV depends on the binding of hemagglutinin-neuraminidase (HN) to α-2,3- and α-2,6-linked sialic acid-containing receptors (38, 39). Pigeons contain abundant α-2,6-linked sialic acid while chickens contain mainly α-2,3-linked sialic acid (40). In this study, α-2,3- and α-2,6-linked sialic acid analogs (3′-sialyllactose and 6′-sialyllactose) were used to investigate the receptor-binding properties of rP0813, rP0713, and rP0506. Both rP0713 and rP0506 exhibited preferential binding affinity for 3′-sialyllactose, rather than 6′-sialyllactose, and the affinity of rP0506 was higher than that of rP0713. By contrast, rP0813 showed a strong binding preference for 6′-sialyllactose, but weak binding to 3′-sialyllactose (Fig. 5A).

To examine whether there were differences in the adsorption capacity of rP0813, rP0713, and rP0506 in chicken-origin cells, DF-1, HD11, and LMH cells were incubated with each virus for 1 h at 4°C to allow viral adsorption but not internalization. Viral RNA was then quantified by real-time RT-PCR. As shown in Fig. 5B, both rP0713 and rP0506 demonstrated significantly higher viral RNA copies than rP0813, with rP0506 exhibiting the highest level. These results indicated the superior adsorption capacity for rP0713 and rP0506, particularly rP0506, across multiple chicken-origin cells. Furthermore, a quantitative internalization assay was performed in DF-1 cells using NDV labeled with two fluorescent dyes, DiOC (green) and R18 (red), as described (41, 42). The virion displayed a red color as the green fluorescence of DiOC was quenched by the high concentration of R18. Following fusion of the viral envelope with the cellular membrane, the two dyes were diluted, the fluorescence of DiOC was no longer quenched by R18, and red and green fluorescence could be detected. Herein, DF-1 cells adsorbed with DiOC/R18-labeled rP0813, rP0713, and rP0506 were further incubated at 37°C for 30 and 60 min to permit internalization. Flow cytometry demonstrated a time-dependent increase in the proportion of DiOC/R18-positive cells in all groups. Furthermore, at each time point, the proportion of DiOC/R18-positive cells was significantly higher in rP0506- and rP0713-infected cells than in rP0813-infected cells, with rP0506 showing the highest proportion (Fig. 5C). These findings indicated that rP0813, rP0713, and rP0506 showed a successively increased capacity for entry into DF-1 cells. Since chicken-origin DF-1 cells contain abundant α-2,3-linked sialic acid (43), receptor binding preference likely leads to differences in cellular entry efficacy among these viruses.

### Investigation of the biological activities *in vitro*

**Fig 6:**
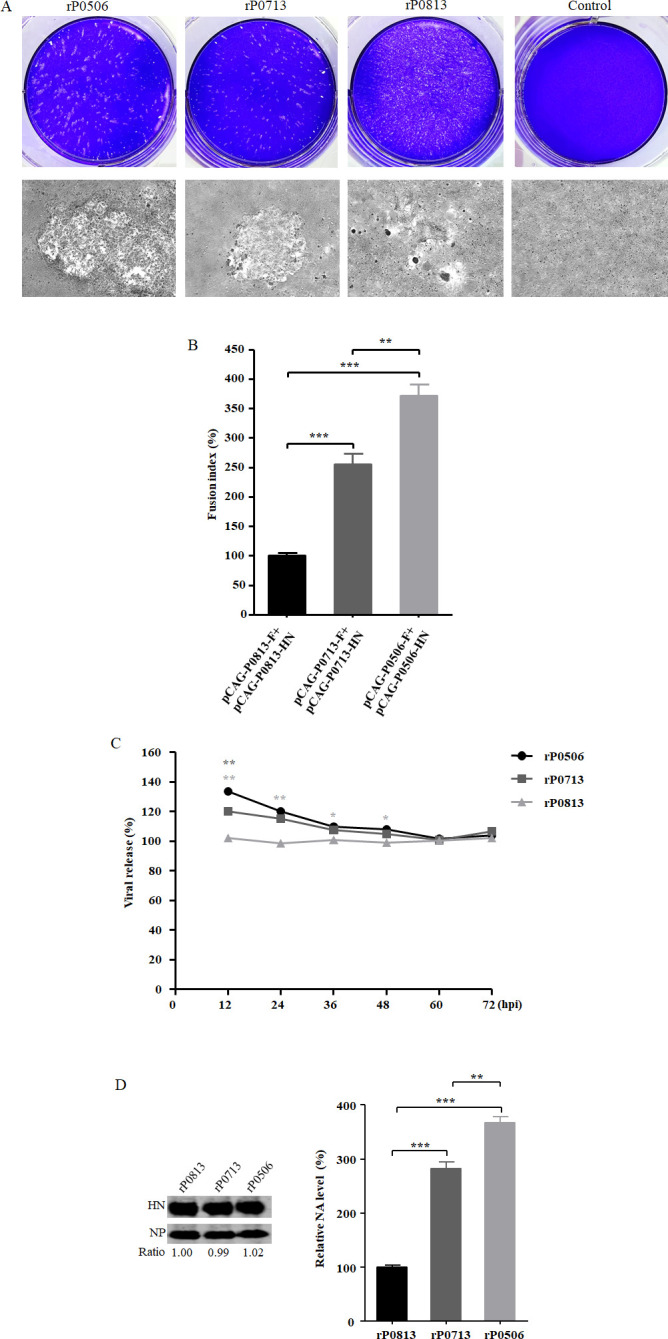
Biological characteristics of rP0813, rP0713, and rP0506 *in vitro*. (**A**) Plaque morphologies induced by rP0813, rP0713, and rP0506. DF-1 cells were inoculated with each virus at an MOI of 0.01. Uninfected SPF chicken embryo AF was used as a negative control. At 36 hpi, the cells were washed and fixed, and cytopathic effects were visualized by crystal violet staining. (**B**) Comparison of the fusion indices of F and HN proteins from rP0813, rP0713, and rP0506. DF-1 cells were co-transfected with plasmids pCAG-P0813-F, -HN, pCAG-P0713-F, -HN, pCAG-P0506-F, and -HN, respectively. At 48 hpt, the cells were stained with a cytoplasmic probe, CellTracker Green CMFDA, and a nuclei dye, 4′,6′-diamidino-2-phenylindole (DAPI). The fusion index was calculated as the ratio of the total number of nuclei to the number of cells in which these nuclei are present, normalized to the cells co-transfected with pCAG-P0813-F and -HN (×100%). (**C**) DF-1 cells were infected with each virus at an MOI of 1. At 12, 24, 36, 48, 60, and 72 hpi, the supernatant and cells were collected, and the HA titers were determined. Viral release was quantified by calculating the ratio of the HA titers in the supernatant to the sum of the HA titers in the supernatant and cells. Viral release efficacy was normalized to that of rP0813 at each time point (×100%). (**D**) Comparison of the NA activities of rP0813, rP0713, and rP0506. Viral proteins were extracted from virus-infected AFs containing 5 log_2_ HAU and subjected to Western blotting for the detection of HN protein using anti-NDV P0713 antiserum. NP protein was used as an internal control. The level of the HN protein was normalized to that of rP0813. NA activity of each virus was determined using a neuraminidase (NA) assay kit and normalized to that of rP0813 (×100%). Data are presented as means ± SD and were analyzed using one-way analysis of variance (*P < 0.05; **P < 0.01; ***P < 0.001).

The replication features of rP0813, rP0713, and rP0506 were further evaluated by plaque formation assay in DF-1 cells. As shown in Fig. 6A, plaque formation was observed in cells infected with all three viruses at 36 hpi, with obvious differences among them. The rP0713 and rP0506 strains yielded obvious plaques, while rP0813 resulted in a small plaque phenotype. The plaque size induced by rP0713 was intermediate between rP0506 and rP0813. Subsequently, the fusion activity mediated by F and HN proteins was evaluated by determining the fusion index after co-transfection of plasmids expressing corresponding proteins from each virus. As shown in Fig. 6B, the fusion index induced by F and HN of rP0713 and rP0506 was dramatically higher than that of rP0813, with rP0506 showing the highest fusion index.

The viral release efficiency of rP0813, rP0713, and rP0506 was also evaluated. Compared to rP0813, both rP0506 and rP0713 exhibited higher viral release efficiency from 12 to 48 hpi, with rP0506 also surpassing rP0713 at 12 hpi (Fig. 6C). Since the release efficiency of progeny NDV is related to the NA activity of the HN protein (44), we determined the NA activity of each virus containing equal amounts of HAU. The NA activity of rP0813, rP0713, and rP0506 exhibited an increasing trend (Fig. 6D). Western blotting confirmed comparable levels of HN protein in virions among the three viruses. These data indicate that the functional activities of F and HN proteins likely contribute to the differential replication phenotypes among these viruses.

### Identification of determinants responsible for the differences in the biological characteristics of viruses

**Fig 7:**
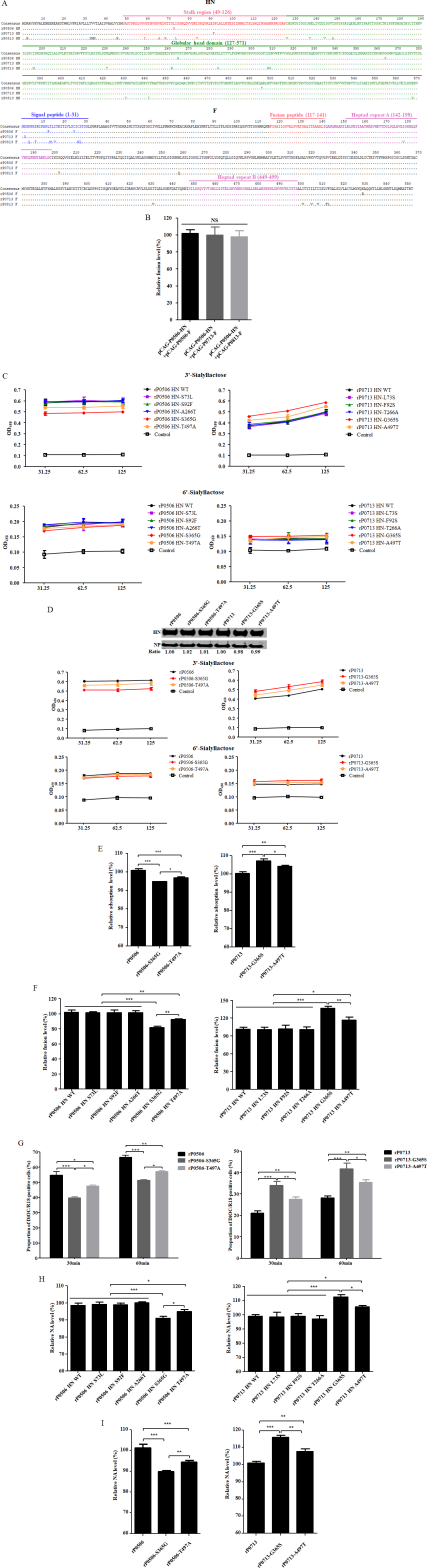
Contribution of HN protein to the differences in biological characteristics of viruses. (**A**) Amino acid sequence alignments of HN and F proteins from rP0713, rP0713, and rP0506. The amino acid residues in each sequence that differ from the consensus sequence were shown. The stalk region and globular head domain in the HN protein, as well as the signal peptide, fusion peptide, and heptad repeat regions in the F protein, were indicated. (**B**) One group of DF-1 cells was incubated with vaccinia virus MVA and then co-transfected with pCAG-P0506-HN and pCAG-P0506-F, pCAG-P0713-F, or pCAG-P0813-F, respectively. Another group of DF-1 cells was transfected with the pOLTV5-luc plasmid. At 6 hpt, cells from each group were collected, mixed, and co-cultured for 48 h. The firefly luciferase activities were measured and presented as the percentages relative to the cells co-transfected with pCAG-P0506-HN + pCAG-P0506-F (100%). (**C**) The binding properties of WT HN and mutants of rP0506 and rP0713 to 3′-sialyllactose-biotin and 6′-sialyllactose-biotin were determined. (**D**) The levels of HN protein in recombinant viruses, rP0506-S365G, -T497A and rP0713-G365S, -A497T, were determined via Western blotting. NP protein was used as an internal control. The level of HN protein of each recombinant virus was normalized to that of the respective parent. The binding properties of recombinant viruses to 3'-sialyllactose-biotin and 6'-sialyllactose-biotin were determined. (**E**) Adsorption capacities of rP0506-S365G, -T497A and rP0713-G365S, -A497T were determined in DF-1 cells. Levels of viral RNA copies were calculated relative to that of the respective parent (×100%). (**F**) Fusion promotion activities of WT HN and mutants of rP0506 and rP0713 were determined as described in (**B**). Activities of each mutant were normalized to that of the respective WT HN (×100%). (**G**) Entry efficacy of rP0506-S365G, -T497A and rP0713-G365S, -A497T was determined via the quantitative internalization assay as described in Fig. 5C. (**H**) NA activities of WT HN and mutants of rP0506 and rP0713 were determined and normalized to that of respective WT HN (×100%). (**I**) NA activities of rP0506-S365G, -T497A and rP0713-G365S, -A497T were determined. Activities of each recombinant virus were normalized to that of the respective parent (×100%). Data are presented as means ± SD and were analyzed using one-way analysis of variance (*P < 0.05; **P < 0.01; ***P < 0.001; NS, no significant difference).

Comparative analysis of the HN and F protein sequences among rP0813, rP0713, and rP0506 revealed multiple amino acid differences. The HN protein of rP0813 exhibited 23 differences relative to rP0713 and rP0506, distributed throughout the protein. Between rP0713 and rP0506, five amino acid differences were identified at positions 73, 92, 266, 365, and 497 in HN, with two in the stalk region (49–126 aa) and three in the globular head domain (127–571 aa) (Fig. 7A). For the F protein, apart from the signal peptide, nine amino acid differences were found in rP0813 when compared with rP0713 and rP0506, while only one difference at position 530 (cytoplasmic tail) was found between rP0713 and rP0506. Of note, the major functional domains of the F protein, including the fusion peptide and the heptad repeat regions A and B, were highly conserved, with a single substitution at position 179 in heptad repeat A of rP0813 (Fig. 7A). Moreover, a luciferase-based fusion assay that co-expressed the HN protein of rP0506 with the F proteins of rP0813, rP0713, and rP0506 demonstrated that the F protein of the three viruses had comparable fusion capacity (Fig. 7B).

These findings suggested that the differences in receptor binding, entry, fusion, and release among rP0813, rP0713, and rP0506 should be attributed to the HN protein. Based on the feasibility of site-directed mutagenesis using reverse genetics and the obvious differences in biological characteristics between rP0713 and rP0506, these two viruses were selected to investigate the effects of differences in the HN protein on viral biological properties. First, receptor binding activity of wild-type (WT) HN proteins from rP0506 and rP0713, along with a series of point mutants, was examined. As shown in Fig. 7C, substitutions at positions 73, 92, and 266 had no significant effects on 3′-sialyllactose binding. Notably, the S365G and T497A substitutions in the rP0506 HN reduced binding affinity to 3′-sialyllactose, whereas the reciprocal substitutions G365S and A497T in the rP0713 HN enhanced it, with the effect of residue 365 being more pronounced. None of the substitutions significantly affected binding of the HN to 6′-sialyllactose. To further validate these findings, a receptor binding assay was performed with recombinant viruses rP0506-S365G, -T497A and rP0713-G365S, -A497T, which contain substitutions at positions 365 and 497 in the HN protein. Western blotting confirmed comparable HN levels in each recombinant virus and its respective parent. The binding assay demonstrated that rP0506-T497A and -S365G exhibited sequentially reduced binding to 3′-sialyllactose, while rP0713-A497T and -G365S showed sequentially increased binding. All recombinant viruses retained 6′-sialyllactose-binding capacity similar to their parents (Fig. 7D). Furthermore, the adsorption capacities of these recombinant viruses in DF-1 cells were determined. As shown in Fig. 7E, compared to the parental rP0506, T497A and S365G substitutions in the HN protein resulted in a sequential reduction in adsorption. Conversely, the A497T and G365S substitutions in the HN protein of rP0713 led to a sequential enhancement of adsorption capacity.

NDV-mediated membrane fusion is coordinated by the HN and F proteins. Since the F proteins of rP0506 and rP0713 exhibited comparable fusion capacity, we assessed the impact of HN substitutions on fusion promotion activity and viral entry. All HN substitutions had no significant effects on protein expression (35). As shown in Fig. 7F, the S365G and T497A substitutions in the HN protein of rP0506 reduced fusion promotion activity, while the G365S and A497T substitutions in the HN protein of rP0713 enhanced it, with residue 365 showing a more pronounced effect. Consistently, a quantitative internalization assay using DiOC/R18-labeled viruses revealed that compared with their parental viruses, rP0506-T497A and -S365G exhibited sequentially decreased entry, whereas rP0713-A497T and -G365S possessed successively increased entry (Fig. 7G).

The effects of substitutions in the HN protein on NA activity were also assessed. Substitutions at positions 365 and 497 in the HN protein significantly affected NA activity, while those at positions 73, 92, and 266 had no notable effects, with the effect at position 365 being more pronounced than at position 497 (Fig. 7H). These effects were further confirmed using recombinant viruses (Fig. 7I). These results collectively revealed that residues at positions 365 and 497 in the HN were responsible for the differential biological characteristics between rP0506 and rP0713, with residue 365 being the key determinant for the superior activities of rP0506.

### Contribution of the viral replication complex to replication capacity

**Fig 8:**
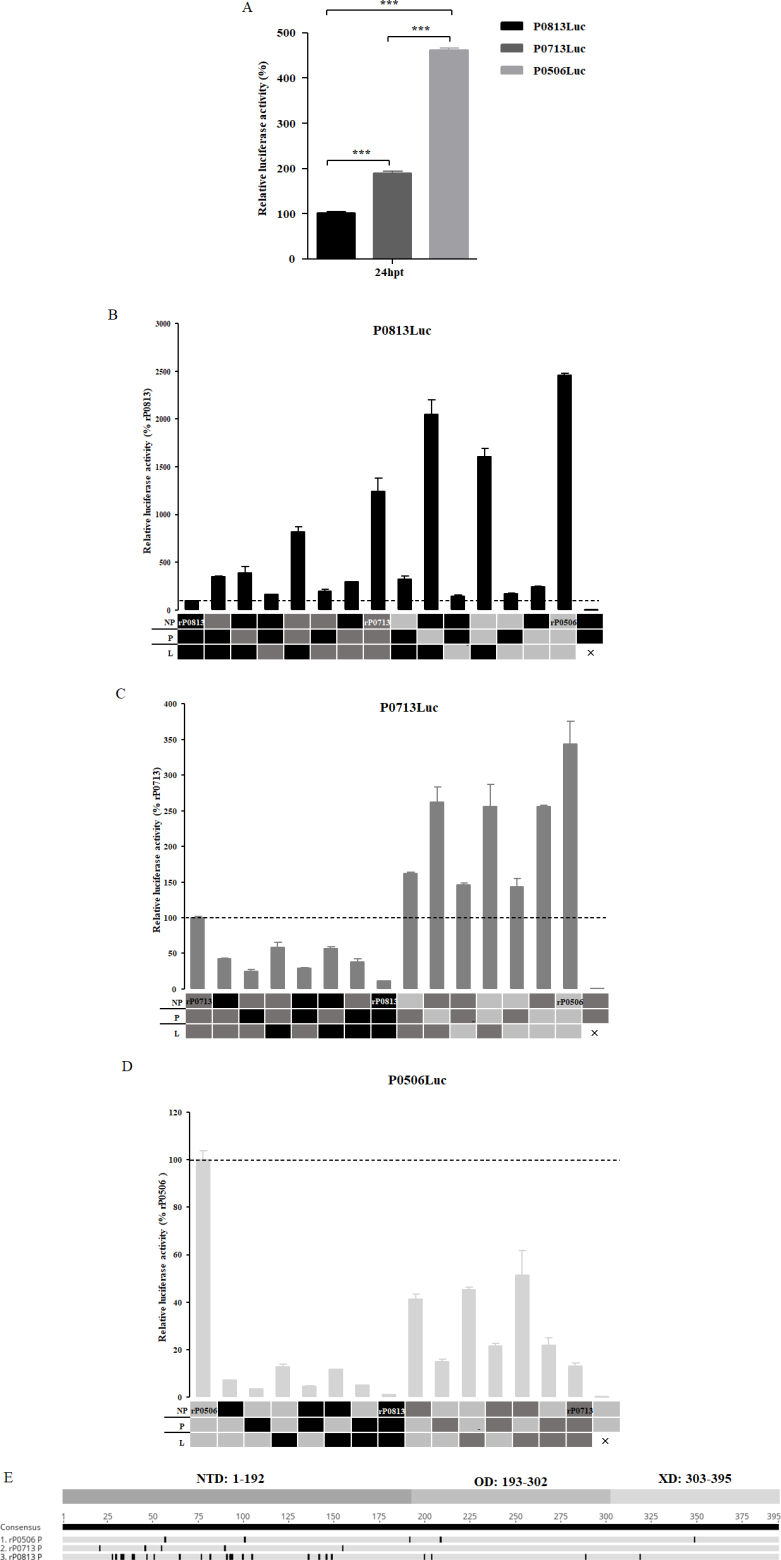
Comparison of viral replication complex activity and evaluation of the contribution of each individual protein. (**A**) Vaccinia virus MVA-infected DF-1 cells were co-transfected with viral minigenome plasmid P0813Luc, P0713Luc, and P0506Luc and corresponding helper plasmids expressing NP, P, and L. Relative luciferase expression levels were normalized to that of P0813Luc (×100%). (**B–D**) Relative luciferase expression levels after co-transfection of MVA-infected DF-1 cells with P0813Luc (**B**), P0713Luc (**C**), and P0506Luc (**D**) and helper plasmids expressing NP, P, and L of rP0813 (black), rP0713 (dark gray), or rP0506 (light gray). The background level of luciferase activity was determined by omitting the L plasmid (**×**). (**E**) Deduced amino acid sequence alignment of P proteins from rP0813, rP0713, and rP0506. Amino acid residues that differ from the consensus sequence in each isolate are shown as black vertical lines. The NDT (1-192), OD (193-302), and XD (303-395) regions in P protein are indicated. Data are presented as means ± SD and were analyzed using one-way analysis of variance (*P < 0.05; **P < 0.01; ***P < 0.001).

To investigate the correlation between the intrinsic activity of the viral replication complex and viral growth characteristics, an *in vitro* replication assay was performed with a minigenome that encodes luciferase in the presence of the NP, P, and L proteins. The replication complex of rP0813 showed significantly lower activity than that of rP0713 and rP0506, with the rP0506 complex exhibiting the highest activity (Fig. 8A). To further evaluate the contribution of individual viral proteins to replication activity, helper plasmids were reciprocally exchanged among rP0813, rP0713, and rP0506, and the replication activity of parental minigenome with heterogeneous replication complex component was determined. For minigenome P0813Luc, replacement with all three helper plasmids from rP0713 or rP0506 significantly enhanced the replication activity, with the highest activity observed using components from rP0506. Individual or pairwise replacement with helper plasmids from rP0713 or rP0506 resulted in increased activity to varying degrees (Fig. 8B). For P0713Luc, individual and combined replacement with helper plasmids from rP0813 significantly reduced replication activity, while either individual or combined replacement with helper plasmids from rP0506 enhanced replication activity (Fig. 8C). For P0506Luc, replacement with the helper plasmids from both rP0813 and rP0713 impaired replication activity, with pronounced effects from rP0813-derived helper plasmids (Fig. 8D). These results revealed that each individual replication complex component had its own contribution to the viral replication capacity. Comparatively, the effects of individual proteins on the replication activity of all three viruses, in descending order, were P, NP, and L. Pairwise replacement of helper plasmids showed that P+NP and P+L combinations induced more significant changes than NP+L. However, no individual or combined replacements achieved the 100% activity level observed with the cognate replication complex. In summary, these results indicated that the intrinsic activity of the viral replication complex was directly related to the replication ability of genotype VI NDVs. Furthermore, although the P protein had the greatest effect on replication capacity, optimal effects required homologous NP and L proteins. Amino acid sequence analysis revealed that differences among P proteins were primarily located in the N-terminal domain (NTD), with only a few variations in the central oligomerization domain (OD) and C-terminal X domain (XD) (Fig. 8E).

### Genomic RNA 5′ terminal trailer region affects viral replication capacity

**Fig 9:**
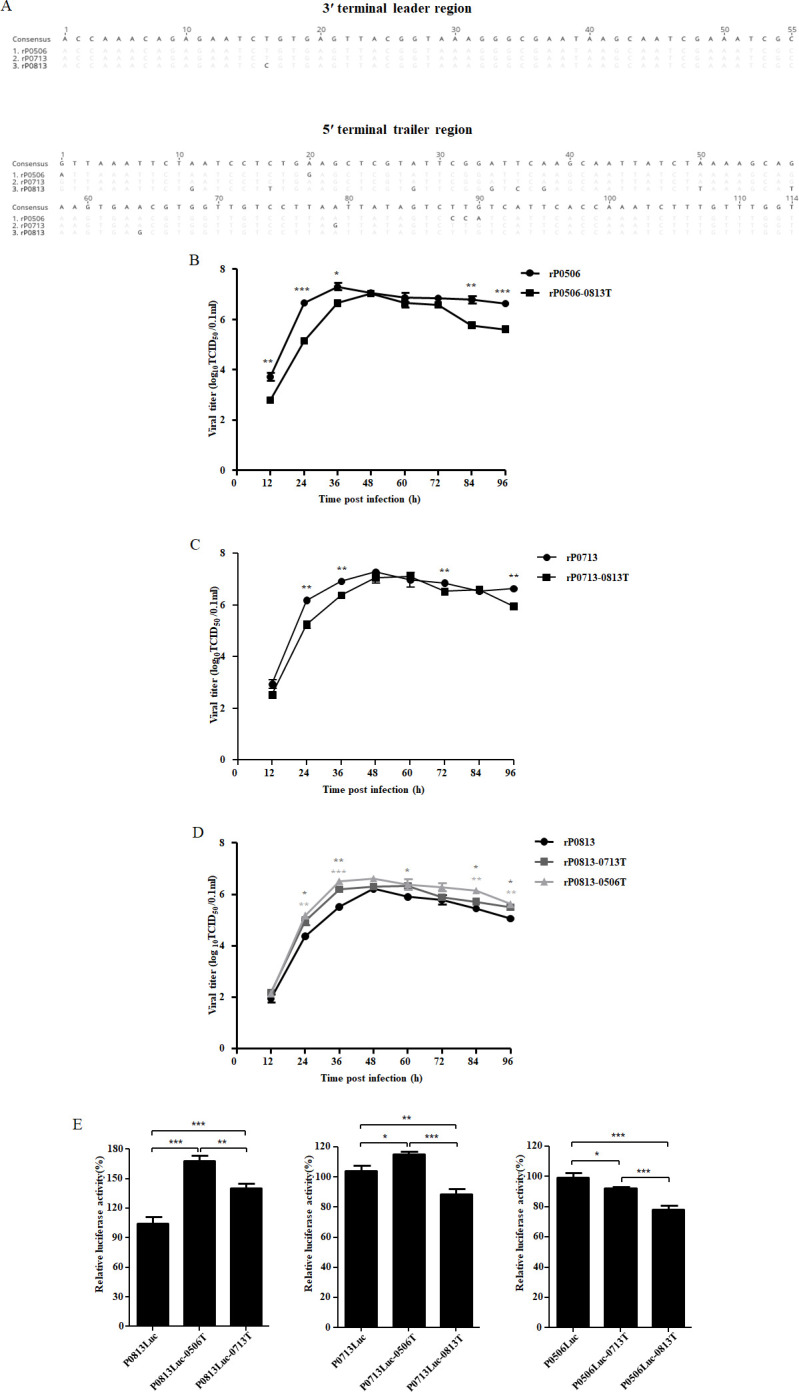
Effects of the RNA 5′ terminal trailer region of the viral genome on the replication of genotype VI NDVs. (**A**) Sequence alignment of the 3′ and 5′ terminal regions from rP0813, rP0713, and rP0506. Nucleotides that differ from the consensus sequence in each isolate are shown. (B–D) Growth kinetics of recombinant viruses with replacement of the 5′ terminal trailer region. DF-1 cells were infected with P0506, rP0506-0813T, rP0713, rP0713-0813T, rP0813, rP0813-0713T, and rP0813-0506T at an MOI of 0.01. Supernatants were collected at 12, 24, 36, 48, 60, 72, 84, and 96 hpi, and viral titers were determined by inoculation at 10-fold dilutions into DF-1 cells and expressed as TCID_50_. Viral titers between P0506 and rP0506-0813T (**B**), between rP0713 and rP0713-0813T (**C**), and among rP0813, rP0813-0713T, and rP0813-0506T (**D**) at each time point were compared. (**E**) Vaccinia virus MVA-infected DF-1 cells were co-transfected with the following minigenome plasmids: P0813Luc, P0813Luc-0713T, P0813Luc-0506T, P0713Luc, P0713Luc-0813T, P0713Luc-0506T, P0506Luc, P0506Luc-0813T, and P0506Luc-0713T, along with their corresponding helper plasmids. Relative luciferase expression levels were normalized to those of the respective parental minigenome plasmid (×100%). Data are presented as means ± SD and were analyzed using Student’s *t*-test or one-way analysis of variance (*P < 0.05; **P < 0.01; ***P < 0.001).

NDV genomic RNA contains a 3′ leader region and a 5′ trailer region, which are essential for transcription and replication of genomic RNA (45). The 3′ leader region sequences of rP0713 and rP0506 were identical, whereas rP0813 differed by a single nucleotide. In contrast, 15 nucleotide differences were identified among the 5′ trailer regions of rP0813, rP0713, and rP0506, nine of which were shared by rP0713 and rP0506 (Fig. 9A). Since both rP0713 and rP0506 exhibited higher replication capacity than rP0813, the 5′ trailer regions of rP0713 and rP0506 were each replaced by that of rP0813, while the rP0813 trailer region was replaced by those of rP0713 and rP0506. Four recombinant viruses, rP0713-0813T, rP0506-0813T, rP0813-0713T, and rP0813-0506T, were recovered. Analysis of growth kinetics demonstrated that during exponential replication (12–36 hpi), rP0713-0813T and rP0506-0813T exhibited significantly lower viral titers than rP0713 and rP0506, respectively (Fig. 9B and C). Conversely, rP0813-0713T and rP0813-0506T showed increased titers relative to rP0813 (Fig. 9D). To further validate the contribution of the 5′ trailer region to the replication capacity of genotype VI NDVs, chimeric minigenomes were constructed by exchanging the trailer region among different strains, and the replication efficiencies were evaluated via an *in vitro* replication assay. The results revealed that chimeric minigenomes P0813Luc-0506T and P0813Luc-0713T, containing the trailer region from rP0506 and rP0713, respectively, showed significantly enhanced replication activity, with P0813Luc-0506T exhibiting higher activity. For P0713Luc, chimeric minigenome P0713Luc-0506T, carrying the trailer region from rP0506, exhibited enhanced replication activity, whereas P0713Luc-0813T, containing the trailer region from rP0813, showed impaired activity. Additionally, chimeric minigenomes P0506Luc-0713T and P0506Luc-0813T, carrying the rP0713 or rP0813 trailer region, exhibited reduced replication activities relative to P0506Luc, with the latter showing the lowest activity (Fig. 9E). Collectively, these findings indicated that the nucleotide composition in the 5′ terminal trailer region contributes to the replication capacity of genotype VI NDVs.

### Comparison of the IFN-antagonistic activity of V proteins

**Fig 10:**
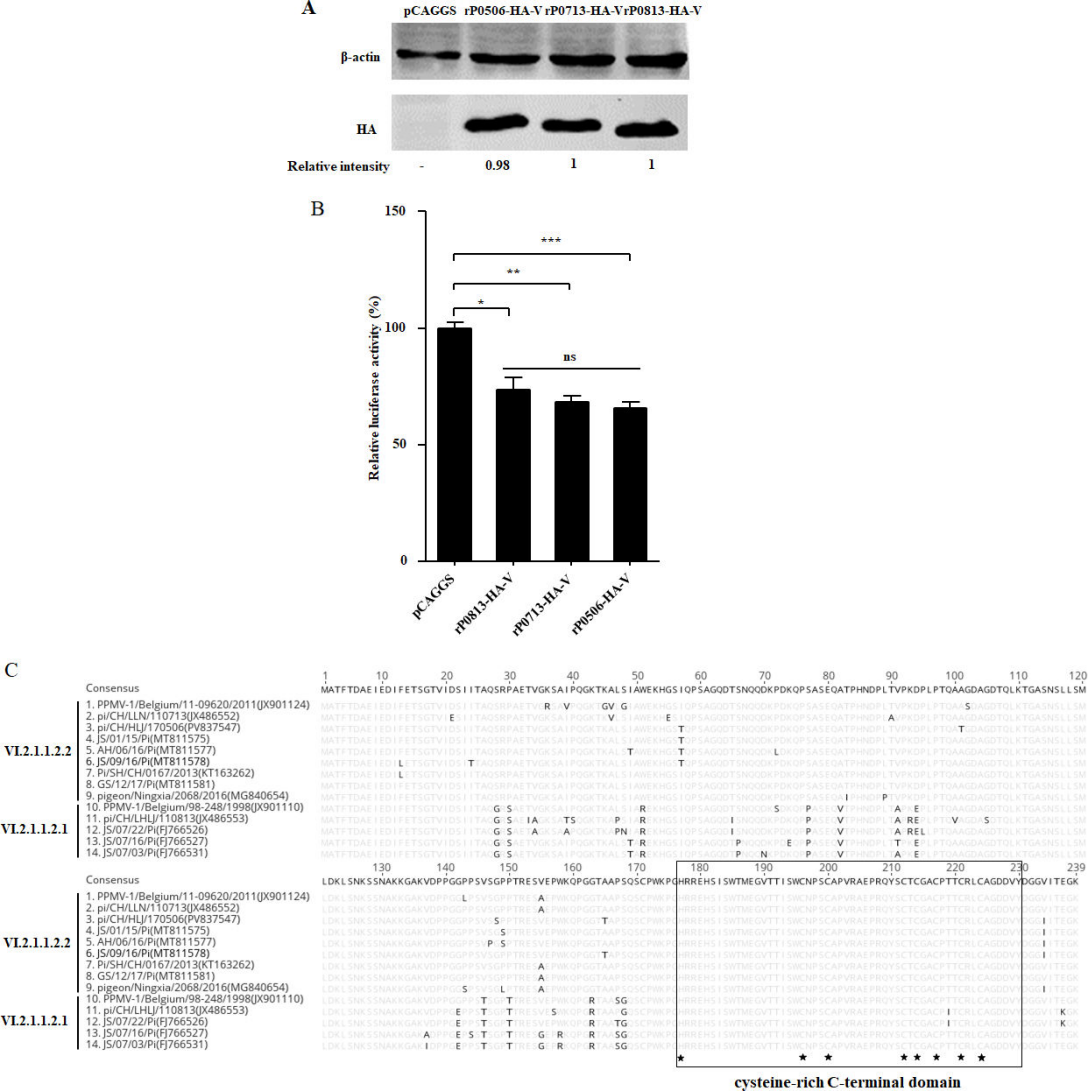
V proteins from different sub-genotype VI NDV strains demonstrate no difference in IFN antagonistic activity. (**A**) DF-1 cells were transfected with V protein-expressing plasmids pCAG-P0813-V, pCAG-P0713-V, pCAG-P0506-V, or pCAGGS vector. Expression of the V protein was determined by Western blotting using an HA tag antibody. β-actin was used as a control. The band intensity of each V protein, relative to β-actin, was determined. Expression levels were normalized to those of rP0813-HA-V. (**B**) Determination of IFN antagonistic activities of V proteins from rP0813, rP0713, and rP0506. DF-1 cells were co-transfected with each V protein-expressing plasmid or pCAGGS vector together with IFN reporter plasmid pChIFN-β-luc and control plasmid pRL-TK. Relative luciferase expression levels after stimulation with poly (I:C) were measured and normalized to cells transfected with the control pCAGGS vector (×100%). (**C**) The amino acid sequence of V proteins from nine VI.2.1.1.2.2 strains and five VI.2.1.1.2.1 strains was aligned. The amino acid residues that differ from the consensus sequence in each isolate are shown. The cysteine-rich CTD, which is responsible for IFN antagonistic activity, is indicated by a black frame, and key amino acid residues are labeled with a black asterisk. Data are presented as means ± SD and were analyzed using one-way analysis of variance (*P < 0.05; **P < 0.01; ***P < 0.001; ns, no significant difference).

NDV V protein is encoded by the P gene via RNA editing and functions as an IFN antagonist (46–48). It has been suggested that the IFN antagonistic activity of the V protein correlates with the virulence and host range of NDV (49, 50). In this study, the IFN antagonistic activities of V proteins from rP0813, rP0713, and rP0506 were evaluated using an IFN-β promoter luciferase reporter assay. Western blotting showed comparable expression level of each V protein in transfected DF-1 cells (Fig. 10A). Compared with the control, luciferase activity was significantly reduced in DF-1 cells expressing the V protein from rP0813, rP0713, and rP0506, with no notable differences observed among the three V proteins (Fig. 10B). The cysteine-rich C-terminal domain (CTD) of the V protein is responsible for its IFN antagonistic activity (51). Sequence alignment of the V proteins from nine sub-genotype VI.2.1.1.2.2 strains and five sub-genotype VI.2.1.1.2.1 strains showed conservation of key residues, including H177, C196, C200, C212, C214, C217, C221, and C224 (Fig. 10C). Collectively, these results demonstrated that the V proteins of rP0813, rP0713, and rP0506 had similar IFN antagonistic activity, suggesting these proteins are unlikely to be related to the virulence and replication capacity of genotype VI NDVs.

## DISCUSSION

Previous assessments have suggested that some genotypes in class II exhibited restricted geographic distributions, while others, such as genotype VI, have spread across continents (2). Here, we show that the currently predominant sub-genotype VI.2.1.1.2.2 first emerged in Belgium and subsequently dispersed across multiple continents, indicating that this sub-genotype has no geographic restrictions. International trade of pigeons likely facilitated this global spread. Notably, an earlier VI.2.1.1.2.2 isolate exhibited thermostability (35), suggesting that this sub-genotype’s resistance to environmental factors (e.g*.,* heat) may contribute to the long-distance transmission, as observed in influenza viruses (52). Furthermore, strains from different countries formed independent clades and accumulated region-unique mutations (Fig. S1 to S3), indicating ongoing local adaptation and evolution of VI.2.1.1.2.2 NDVs. A recent ND outbreak caused by VI.2.1.1.2.2 NDVs in laying hens in Switzerland has been reported (24). The Chinese VI.2.1.1.2.2 strain P0506 showed higher replication capacity and pathogenicity in chickens. These results demonstrate that the evolution of VI.2.1.1.2.2 NDVs has increased the risk for chickens and highlighted the emergence of NDV variants with pandemic potential.

Global phylogenetic analysis suggested that the two prevalent sub-genotypes, VI.2.1.1.2.1 and VI.2.1.1.2.2, may originate from respective ancestors in Belgium. One route of transcontinental viral transmission of virus is likely via wild bird migration (53). Interhost transmission of genotype VI NDVs among pigeons and wild birds has been reported (20). However, the different migratory flyways between China and Belgium suggest that the spread of genotype VI NDVs from Belgium to China through migratory birds is unlikely. Instead, given Belgium’s prominence in pigeon racing and breeding, and China’s growing pigeon industry (54, 55), the pigeon trade appears to be a plausible introduction pathway of Chinese VI. 2.1.1.2.1 and VI. 2.1.1.2.2 NDVs. Human activities, including extensive racing, exhibitions, or the trade of pigeons, likely facilitate the spread of these viruses in China (6). The circulation of genotype VI NDVs in wild birds indicates that the role of wild birds, especially migratory birds, in the dispersal of genotype VI NDVs in some areas cannot be ruled out (6, 20, 56). Moreover, the dominance of VI. 2.1.1.2.2 NDVs may also indicate their superior adaptation and replication efficiency in birds.

Pathogenicity evaluations of genotype VI NDVs in different bird species, including pigeons and chickens, have been documented. To summarize, despite marked differences in clinical manifestations, genotype VI NDVs exhibit broad tissue distribution and similar viral shedding patterns in both pigeons and chickens. In contrast, higher viral loads are generally observed in tissues and swab samples from pigeons (13, 57–60). In this study, we demonstrated that the replication capacity and pathogenicity of rP0813, rP0713, and rP0506 increased progressively in chickens. Our findings, combined with previous reports, suggest that viral replication level is directly associated with the pathogenic phenotype of genotype VI NDVs in chickens (17, 60). Another concern is viral shedding via oral and cloacal routes in infected chickens exhibiting minimal or mild clinical symptoms (13, 59, 60), which may facilitate viral circulation in poultry flocks and pose a transmission risk.

Analysis of differences across various stages of the viral life cycle revealed that compared with rP0813, rP0713 and rP0506 exhibited progressively increasing efficiencies in α-2,3-linked sialic acids binding, entry, cell-cell fusion, genome replication, and release. Except for genome replication, all other processes were directly associated with the HN and F proteins’ function. Herein, sequence analysis of the F protein revealed that the variable residues were primarily located in the signal peptide, while functional domains in the F protein were highly conserved among rP0813, rP0713, and rP0506, and all three F proteins exhibited similar fusion capabilities. Therefore, substitutions in the signal peptide of the F protein were unlikely to significantly affect replication or virulence of genotype VI NDVs. The observed differences in plaque size among these strains are therefore likely due to differences in the fusion-promoting activity of their HN proteins. Regarding the release efficiency, differences were evident at the early stage of infection. Replication of rP0813, rP0713, and rP0506 within cells reached a plateau at the late stage of infection (after 48 hpi), due to severe cell damage. During this stage, all infected cells have reached maximum virus yield and completed release of mature virions, resulting in no significant differences in release efficiency. Viral release during the early stages of replication facilitates viral dissemination and spread, thereby enhancing viral replication and pathogenicity. Collectively, these findings indicate that HN, rather than F, is the determinant responsible for the differences in biological characteristics among these viruses.

Further investigation using rP0713 and rP0506 as models identified residues 365 and 497 in the HN globular head domain as determinants of differences in viral biological characteristics, with position 365 being the key residue. Compared to G365 and A497 in rP0713 HN, S365 and T497 in rP0506 HN have a greater propensity to form hydrogen bonds with the ligand (61), suggesting that the S365 and T497 residues could confer greater binding affinity to sialic acid receptors. Moreover, this potent binding may more effectively induce conformational changes in the HN protein, thereby enhancing its fusion-promoting activity (62). The enhanced ligand affinity should also explain the increased NA activity and viral release efficiency. Furthermore, although both serine (S) and threonine (T) are hydrophilic polar amino acids, the side chain of S has less steric hindrance than T, potentially favoring protein structural flexibility and catalytic function (61), these are likely the reasons for the greater functional impact of S365. Simultaneous alterations of receptor binding, fusion promotion, and NA activities induced by specific residue substitutions in HN have been reported in NDV (63, 64). However, due to the considerable number of divergent amino acids between the HN proteins of the VI.2.1.1.2.1 strain (P0813) and the VI.2.1.1.2.2 strains (P0713 and P0506), the significance of these divergent sites was not evaluated in the present study. Thus, the key residues influencing HN function and viral phenotype of VI.2.1.1.2.1 NDV warrant further investigation.

The contributions of viral RNA replication-associated proteins and genomic 3′ and 5′ terminal regions to the replication and virulence of the chicken-origin genotype II NDV have been evaluated. The L protein was considered the major contributor to viral replication, while the 3′ leader and 5′ trailer sequences showed no significant effects (36, 65, 66). Here, we showed that in genotype VI NDVs, each component of the replication complex influenced viral replication efficiency, with the P protein being the most potent contributor. Furthermore, exchanging the 5′ trailer region markedly altered the replication complex activity and viral replication. These findings implied that modulation of viral replication by the P protein and the 5′ terminal trailer sequence might be unique features of genotype VI NDVs, suggesting genotype-dependent roles of viral factors in NDV replication. During the life cycle of NDV, the P protein binds the nascent NP chain via its NTD domain, directing specific encapsidation of the nascent viral RNA trailer sequence and further promoting progeny genome synthesis by using the trailer sequence as a promoter (67, 68). In addition, the nucleotide sequences within the 5′ trailer region could also affect affinity for the NP protein and its promoter function (69, 70). Taken together, we speculated that the differences in the NTD of the P protein and the 5′ terminal trailer region might synergistically affect the efficiency of encapsidation of nascent viral RNA, thereby altering progeny genome synthesis and replication efficiency of genotype VI NDVs. The significance of the specific differences in the P protein and the 5′ trailer region in the replication of genotype VI NDVs should be amenable to investigation by reverse genetics in the future.

The non-structural protein V is encoded by the NDV P gene via RNA editing. Some studies have suggested that the IFN antagonistic activities of V proteins are correlated with the virulence properties of NDVs (50, 71), but another study indicated that the V protein was not directly related to the virulence difference between two genotype II NDV strains (65). In this study, V proteins from rP0813, rP0713, and rP0506 showed comparable expression levels in DF-1 cells and exhibited similar IFN antagonistic activities. The CTD of the V protein, which mediates IFN antagonism, was converted between sub-genotypes VI.2.1.1.2.1 and VI.2.1.1.2.2 NDVs, suggesting that the V protein might not be involved in virulence differences within genotypes VI NDVs.

Our results showed that the differences in biological characteristics of VI.2.1.1.2.1 and VI.2.1.1.2.2 NDVs were attributed to multiple viral factors, including the HN protein, the viral replication complex (NP, P, and L), and the genomic RNA 5′ trailer region. Among these, specific residues in the HN protein of the prevalent VI.2.1.1.2.2 NDV (rP0506) were identified as key determinants responsible for its superior performance at multiple stages of the virus life cycle, especially invasion efficacy. Therefore, the HN protein should be considered as the primary factor determining viral replication capability and virulence. Additionally, the synergistic effect of the P protein and the 5′ trailer region in viral genome replication was identified as the secondary contributor. Of particular note, the preferential binding affinity for α-2, 3-linked sialic acid and increased replication and shedding in chickens indicate that rP0506-like VI.2.1.1.2.2 NDVs may pose a potential threat to chicken flocks.

## MATERIALS AND METHODS

### Viruses, cells, eggs, and animals

Three genotype VI NDV strains, P0813, P0713, and P0506, were used in this study (34, 35). A stock of each virus was prepared by inoculating 9-day-old SPF embryonated chicken eggs once, and the viral titers were determined via limiting dilution on 9-day-old SPF embryonated chicken eggs and expressed as the 50% embryo infectious dose (EID_50_) using the method of Reed and Muench (72). The modified vaccinia virus MVA, expressing T7 RNA polymerase, was propagated as described (73). DF-1, LMH, and BSR-T7/5 cells were maintained in Dulbecco’s modified Eagle’s medium (DMEM) containing 10% fetal bovine serum (FBS). HD11 cells were cultured in RPMI 1640 medium supplemented with 10% FBS. SPF embryonated chicken eggs and chicks were obtained from the National Poultry Laboratory Animal Resource Center, Harbin Veterinary Research Institute, Chinese Academy of Agricultural Sciences (HVRI, CAAS). The animal experimental protocol was approved by the Animal Welfare Committee of HVRI (IACUC-210907-02). All experiments were conducted at appropriate biosafety levels.

### Sequence and phylogenetic analyses

The complete F gene CDS sequences of all available genotype VI NDV strains with submission dated up to 8 April 2025 (*n* = 412) were collected. Among them, 265 genotype VI NDV F CDS sequences submitted before 21 February 2019 were obtained from the F gene data set established by Dimitrov et al. in 2019 (3). Another 147 genotype VI NDV F CDS sequences of NDV strains submitted from 21 February 2019 to 18 April 2025 were downloaded from the GenBank database. Sequence identity and phylogenetic analysis were performed with Geneious Prime software and MEGA 10 software, respectively (74, 75). A phylogenetic tree was constructed using the maximum likelihood method in MEGA 10 with 1,000 bootstrap replicates. Metadata, including year and location of isolations, were collected from annotations in the GenBank database and used for temporal and geographic distribution analysis.

### Plasmid construction

Construction of the plasmid containing the full-length cDNA of P0813 based on the transcription vector pOLTV5 was performed as previously described (37, 76). Briefly, the genomic 3′/5′ terminal regions were first constructed as a chimera containing a PmeI restriction site and cloned into pOLTV5. Then, four genomic fragments were successively cloned into the plasmid constructed in the previous step using the In-Fusion Cloning Kit (Takara, Dalian, China). The obtained plasmid containing the full-length cDNA of P0813 was designated as P0813FL. The plasmids P0713FL and P0506FL containing the full-length P0713 and P0506 cDNA were previously constructed (35). In addition, the genomic trailer regions of P0713 and P0506 were replaced by that of P0813, and the corresponding region of P0813 was replaced by that of P0713 and P0506, respectively. The resulting plasmids were designated as P0713FL-0813T, P0506FL-0813T, P0813FL-0713T, and P0813FL-0506T, respectively.

For the construction of minigenome plasmids, the leader and trailer regions of P0813, P0713, and P0506 were amplified from plasmids P0813FL, P0713FL, and P0506FL, respectively. The CDS of the firefly luciferase gene with an additional termination codon (TAA) was amplified from the pGL3-Basic vector (Promega, Madison, WI, USA). The chimeras containing the luciferase CDS flanked at the 5′ side by the trailer region and at the 3′ side by the leader region of P0813, P0713, and P0506 were constructed via overlap PCR and cloned into the pOLTV5 vector under control of the T7 promoter, resulting in three minigenome plasmids designated P0813Luc, P0713Luc, and P0506Luc, respectively. In addition, the CDS of luciferase was amplified from the pGL3-Basic vector and directly cloned into the pOLTV5 vector under the control of the T7 promoter, resulting in the plasmid designated pOLTV5-luc. In addition, the genomic 5′ terminal trailer region of each minigenome plasmid was replaced with that of the other two plasmids. The resulting plasmids were designated as P0813Luc-0713T, P0813Luc-0506T, P0713Luc-0813T, P0713Luc-0506T, P0506Luc-0813T, and P0506Luc-0713T, respectively.

The CDS of NP, P, and L genes, and F and HN genes of P0813, P0713, and P0506 were amplified from plasmids P0813FL, P0713FL, and P0506FL and cloned into the pCI-neo vector and pCAGGS vector, respectively (77). The resultant plasmids were named pCI-P0813-NP, -P, -L, pCAG-P0813-F, -HN, pCI-P0713-NP, -P, -L, pCAG-P0713-F, -HN, pCI-P0506-NP, -P, -L, pCAG-P0506-F, and -HN, respectively. In addition, plasmids pCAG-P0506-HN-S73L, -S92F, -A266T, -S365G, -T497A, pCAG-P0713-HN-L73S, -F92S, -T266A, -G365S, and -A497T, which express rP0506 and rP0713 HN mutants with amino acid substitutions at positions 73, 92, 266, 365, and 497 in HN protein, were constructed in our previous study and were used (35).

To construct the HA-tagged V protein expressing plasmid, the CDS of the HA tag was introduced after the initial codon of the P gene, and site-directed mutagenesis was performed on pCAG-P0813-P, pCAG-P0713-P, and pCAG-P0506-P to introduce one nontemplate G residue at the RNA editing site through overlap PCR (78). The resulting plasmids were designated pCAG-P0813-V, pCAG-P0713-V, and pCAG-P0506-V, respectively. The chicken IFN-β promoter sequence was cloned into the pGL3-Basic vector to construct an IFN reporter plasmid designated pChIFN-β-luc as described (50).

### Rescue and characterization of viruses

To rescue the virus, BSR T7/5 cells were co-transfected with plasmids expressing NP, P, and L together with plasmids containing the full-length cDNA clone as previously described (37, 79). After 3 days, the culture supernatant and cells were harvested and inoculated into the allantoic cavities of 9-day-old SPF embryonated eggs. The AF was collected to confirm the presence of NDV particles using HA and HI assays. The recombinant viruses derived from plasmids P0813FL, P0713FL-0813T, P0506FL-0813T, P0813FL-0713T, and P0813FL-0506T were named rP0813, rP0713-0813T, rP0506-0813T, rP0813-0713T, rP0813-0506T, respectively. After three successive passages in SPF embryonated eggs, virus stock of each rescued virus was prepared, and the viral titer was determined in SPF eggs as described previously (37, 72, 80). Viral RNA was extracted from the stocks of recovered viruses, and the genomic sequences of recombinant viruses were determined. The swapped trailer regions in chimeric rP0713-0813T, rP0506-0813T, rP0813-0713T, and rP0813-0506T were also sequenced. Recombinant viruses, rP0713 and rP0506, as well as rP0713-G365S, -A497T, rP0506-S365G, and -T497A, which contain amino acid substitutions at positions 365 and 497 in the HN protein, have been recovered in our previous work (35). To evaluate the levels of HN protein in virions of rP0813, rP0713, rP0506, rP0713-G365S, -A497T, rP0506-S365G, and -T497A, viral proteins were extracted from AFs infected with these viruses, containing 5 log_2_ HAU. The protein samples were subjected to SDS-PAGE and Western blotting using anti-NDV P0713 antiserum to evaluate the level of HN protein. The NP protein was detected with anti-NP antibody to normalize the expression level of the HN protein (79).

### Viral growth kinetics *in ovo* and *in vitro*

For the determination of growth kinetics of the indicated virus *in ovo*, 9-day-old SPF embryonated chicken eggs were inoculated with 100 μL of 100 EID_50_ of each virus. AFs from five eggs receiving each virus were harvested at 12, 24, 36, 48, 60, and 72 hpi, and the viral titers were determined in SPF eggs using the above-described EID_50_ method.

The growth kinetics of the indicated virus *in vitro* were determined in DF-1 cells. Cells in six-well plates were infected with each virus at an MOI of 0.01 and incubated with DMEM containing 5% FBS at 37°C. The supernatants were collected at 12-h intervals until 72 h after inoculation. The viral titers were determined by inoculation at 10-fold dilutions into DF-1 cells and expressed as median tissue culture infectious dose (TCID_50_) using the endpoint method of Reed and Muench (72).

### Pathogenicity and replication in chickens

Sixty 4-week-old SPF White Leghorn chicks were randomly divided into four groups of 15 birds each. Chicks in groups 1–3 were inoculated with rP0813, rP0713, and rP0506 via the oculonasal route, with a dose of 10^6^ EID_50_/100 μL per chick, respectively. Chicks in group 4 were inoculated with sterile AFs. Clinical signs were recorded daily until 14 dpi. Oral and cloacal swabs were collected from surviving birds in each group at 4, 7, 10, and 14 dpi. Five birds from each group were sacrificed at 4 dpi, gross lesions were examined, and tissue samples of the brain, trachea, lung, proventriculus, spleen, cecal tonsil, kidney, and intestine were collected. Tissue samples of the brain, trachea, and intestine were divided into two parts: one to determine viral load, and the second was fixed in 10% buffered formalin for histopathology examination. The viral load in swabs and tissue samples was determined via real-time RT-PCR. Samples with a Ct value <35 were considered as NDV positive (58). In addition, serum samples collected from birds in all groups at 4, 7, 10, and 14 dpi were subjected to antibody detection via HI assay.

### Solid-phase direct receptor binding assay

The receptor-binding property of NDVs was assessed using a solid-phase direct binding assay as described previously (81). Briefly, 3′-sialyllactose-biotin and 6′-sialyllactose-biotin (125, 62.5, and 31.25 ng) were used to coat Pierce streptavidin high-binding capacity coated 96-well plates (Thermo Fisher Scientific, Waltham, MA, USA) overnight at 4°C. The free binding sites were blocked with 5% skim milk at 37°C for 1 h. Next, 50 µL of 5 log_2_ HAU of NDVs diluted in 2% skim milk was added and incubated overnight at 4°C. Meanwhile, 50 µL of 5 log_2_ HAU of the WT HN proteins of rP0713 and rP0506, as well as HN-L73S, -F92S, -T266A, -G365S, -A497T mutants of rP0713 and HN-S73L, -S92F, -A266T, -S365G, -T497A mutants of rP0506, obtained in our previous study, were also added and incubated (35). Uninfected SPF chicken embryo AF was used as a negative control. Anti-NDV P0713 antiserum (1:100) was added, followed by incubation at 4°C for 6 h. Horseradish peroxidase-conjugated rabbit anti-chicken antibody was added and incubated at 4°C for 2 h, then the plates were developed with tetramethylbenzidine substrate solution. The reaction was stopped with 0.5 M H_2_SO_4_, and the absorption was measured at 450 nm.

### Adsorption and internalization assays

Adsorption and internalization assays were performed as described (38, 82). Briefly, for the adsorption assays of rP0813, rP0713, and rP0506, three kinds of chicken-derived cells, DF-1, HD11, and LMH cells, cultured in six-well plates, were inoculated with each virus at an MOI of 10 for 1 h at 4°C to allow for virus adsorption. After three washes with cold PBS, cells were harvested and submitted for viral RNA extraction and quantification of viral load via real-time RT-PCR assay (79, 83). Moreover, the adsorption assay of the recombinant viruses rP0713-G365S, -A497T, rP0506-S365G, and -T497A in DF-1 cells was performed following the same procedure.

For the internalization assay, rP0813, rP0713, rP0506, rP0713-G365S, -A497T, rP0506-S365G, and -T497A were labeled with 3.3 μM DiOC (green fluorescence) and 6.7 μM R18 (red fluorescence) fluorescent (Thermo Fisher Scientific) as described (41, 42, 82). DF-1 cells were inoculated with each DiOC/R18-labeled virus at an MOI of 10 for 1 h at 4°C. After washing with cold PBS, the cells were incubated with pre-warmed DMEM for 30 and 60 min at 37°C for internalization. Next, the cells were washed with PBS and treated with citrate buffer (pH 3) to remove noninternalized viruses. Then, the cells were fixed in 4% paraformaldehyde and subjected to flow cytometric analysis. The proportion of green- and red-double positive cells in 10,000 cells was measured with the Apogee A60-Universal flow cytometer (Apogee Flow Systems Ltd, Hemel Hempstead, UK).

### Plaque and fusogenic ability evaluation

For plaque assays, DF-1 cells in six-well plates were inoculated with rP0813, rP0713, or rP0506 at an MOI of 0.01. Uninfected SPF chicken embryo AF was used as a negative control. After adsorption, the cells were washed with PBS and cultured in DMEM containing 5% FBS at 37°C for 36 h. The cell monolayers were washed, fixed with 4% paraformaldehyde, and stained with crystal violet to visualize plaques.

The fusogenic ability of the F protein from rP0813, rP0713, and rP0506 was evaluated using the fusion index. DF-1 cells were co-transfected with plasmids expressing F and HN proteins of rP0813, rP0713, and rP0506. At 48 h post-transfection (hpt), the cells were washed with PBS and stained with a cytoplasmic probe, CellTracker Green CMFDA (Thermo Fisher Scientific) according to the manufacturer’s instructions. After washing with PBS, the cells were fixed with paraformaldehyde and stained with DAPI. Cells were visualized using an inverted fluorescence microscope. Nuclei and cells were counted by observing 20 fields per virus. The fusion index was defined as the ratio of the total number of nuclei to the number of cells in which these nuclei are present (66). Furthermore, the fusion promotion activity of the WT HN and mutants of rP0713 and rP0506 was quantitatively evaluated by a luciferase reporter assay. Specifically, one group of DF-1 cells, which were pre-inoculated with vaccinia virus MVA at an MOI of 1, was co-transfected with F and WT HN or HN mutants expression plasmids from rP0713 or rP0506. Meanwhile, another group of DF-1 cells was co-transfected with plasmids pOLTV5-luc and pRL-TK, which contain the *Renilla* luciferase gene (Promega) for normalization. At 6 hpt, two groups of cells were digested with 0.53 mM EDTA and diluted to equal concentration with DMEM containing 10% FBS. Then, 100 μL of cells from each group were mixed and co-cultured in 96-well cell plates for 48 h. The firefly luciferase activities were measured using the Dual-luciferase reporter assay system (Promega) according to the manufacturer’s instructions.

### NA assay

The NA activities of NDVs and HN proteins were examined using a Neuraminidase Assay Kit (Beyotime, Shanghai, China) as previously described (84). Briefly, AFs infected with NDV or HN proteins, which contain 5 log_2_ HAU viruses or proteins, were mixed with 70 μL detection buffer, followed by the addition of 10 μL NA fluorogenic substrate and 10 μL double-distilled water. Uninfected AF was used as a negative control. Following incubation for 30 min at 37°C, the fluorescence was measured at 450 nm using a multifunctional microplate reader (PerkinElmer, Waltham, MA, USA).

### Replication efficiency assay

DF-1 cells were infected with vaccinia virus MVA at an MOI of 1 for 1 h. Subsequently, the cells were co-transfected with minigenome plasmids P0813Luc, P0813Luc-0713T, P0813Luc-0506T, P0713Luc, P0713Luc-0813T, P0713Luc-0506T, P0506Luc, P0506Luc-0813T, or P0506Luc-0713T and helper plasmids expressing NP, P, and L originating from each parental virus. For normalization, the plasmid pRL-TK was co-transfected (Promega). In addition, minigenome plasmids P0813Luc, P0713Luc, and P0506Luc were co-transfected with three helper plasmids in which one or two of the plasmid(s) from the other two viruses. Following incubation for 24 h, the levels of luciferase activity were measured as described above. The values are indicated as the percentages of luciferase activity for cells co-transfected with minigenome and helper plasmids from the parental virus (100%).

### Virulence assays

Virulence of NDVs was evaluated through two standard pathogenicity tests used for NDV, the MDT in 9-day-old embryonated SPF chicken eggs and the ICPI in 1-day-old SPF chicks, as described (85).

### Viral release assay

The release efficiency of NDVs was examined by determining the HA titers in the supernatant and cells as previously described (86). DF-1 cells cultured in 12-well plates were infected with each virus at an MOI of 1, and then incubated with 1 mL DMEM supplemented with 5% FBS at 37°C. Following the collection of supernatants at the indicated time points, the cells were digested via trypsinization and resuspended in 1 mL DMEM containing 5% FBS. The viral release efficiency was quantified by calculating the ratio of the HA titers in the supernatant to the total titers in the supernatant and cells.

### IFN antagonistic activity

DF-1 cells were transfected with V protein-expressing plasmids pCAG-P0813-V, pCAG-P0713-V, pCAG-P0506-V, or pCAGGS vector, and expression of HA-tagged-V protein was determined by Western blotting using the HA tag antibody. Then, DF-1 cells were co-transfected with each of the V protein-expressing plasmids or pCAGGS vector together with IFN reporter plasmid pChIFN-β-luc and control plasmid pRL-TK. Following incubation for 24 h, the cells were stimulated with poly (I:C) and cultured for 16 h. Luciferase activity was measured with a luciferase reporter assay kit as described above.

### Statistical analysis

Data are expressed as means ± SD and were analyzed using GraphPad Prism software (GraphPad Software, La Jolla, CA, USA). Pairwise comparisons were analyzed by Student’s *t*-test. Multigroup comparisons were conducted by one-way analysis of variance with Bonferroni’s correction. Differences were considered significant if *P* values were < 0.05.

## Data Availability

All primer sequences used for plasmid construction and mutations are available on request. The sequences used in the phylogenetic analyses have been deposited in Genbank. All accession numbers of the sequences are provided in Table S1.

## SUPPLEMENTAL MATERIAL

Figure S1 jvi.01799-25-s0001.pdf Phylogenetic analysis of genotype VI NDVs.

Figure S2 jvi.01799-25-s0002.pdf Comparison of nucleotide of F gene CDS among sub-genotype VI.2.1.1.2.2 NDVs isolated from all countries except China.

Figure S3 jvi.01799-25-s0003.pdf Comparison of nucleotide of F gene CDS among sub-genotype VI.2.1.1.2.2 NDVs isolated from China.

Figure S4 jvi.01799-25-s0004.pdf Comparison of deduced amino acid sequences of F protein among sub-genotype VI.2.1.1.2.2 NDVs isolated from China.

Table S1 jvi.01799-25-s0005.xlsx Details of genotype VI NDV isolates used for phylogenetic analysis.

ASM does not own the copyrights to Supplemental Material that may be linked to, or accessed through, an article. The authors have granted ASM a non-exclusive, world-wide license to publish the Supplemental Material files. Please contact the corresponding author directly for reuse.
