# Supplementary material for: Global evolution dynamics of genotype VI NDVs and dissection of the biological properties of strains from the prevalent sub-genotypes
Source: J Virol. 2025 Dec 30;100(2):e01799-25. doi: 10.1128/jvi.01799-25 (PMC12911885; doi:10.1128/jvi.01799-25)
Supplement: Figure S2 — Comparison of nucleotide of F gene CDS among sub-genotype VI.2.1.1.2.2 NDVs isolated from all countries except China. [file jvi.01799-25-s0002.pdf]

Supplemental Figure 2

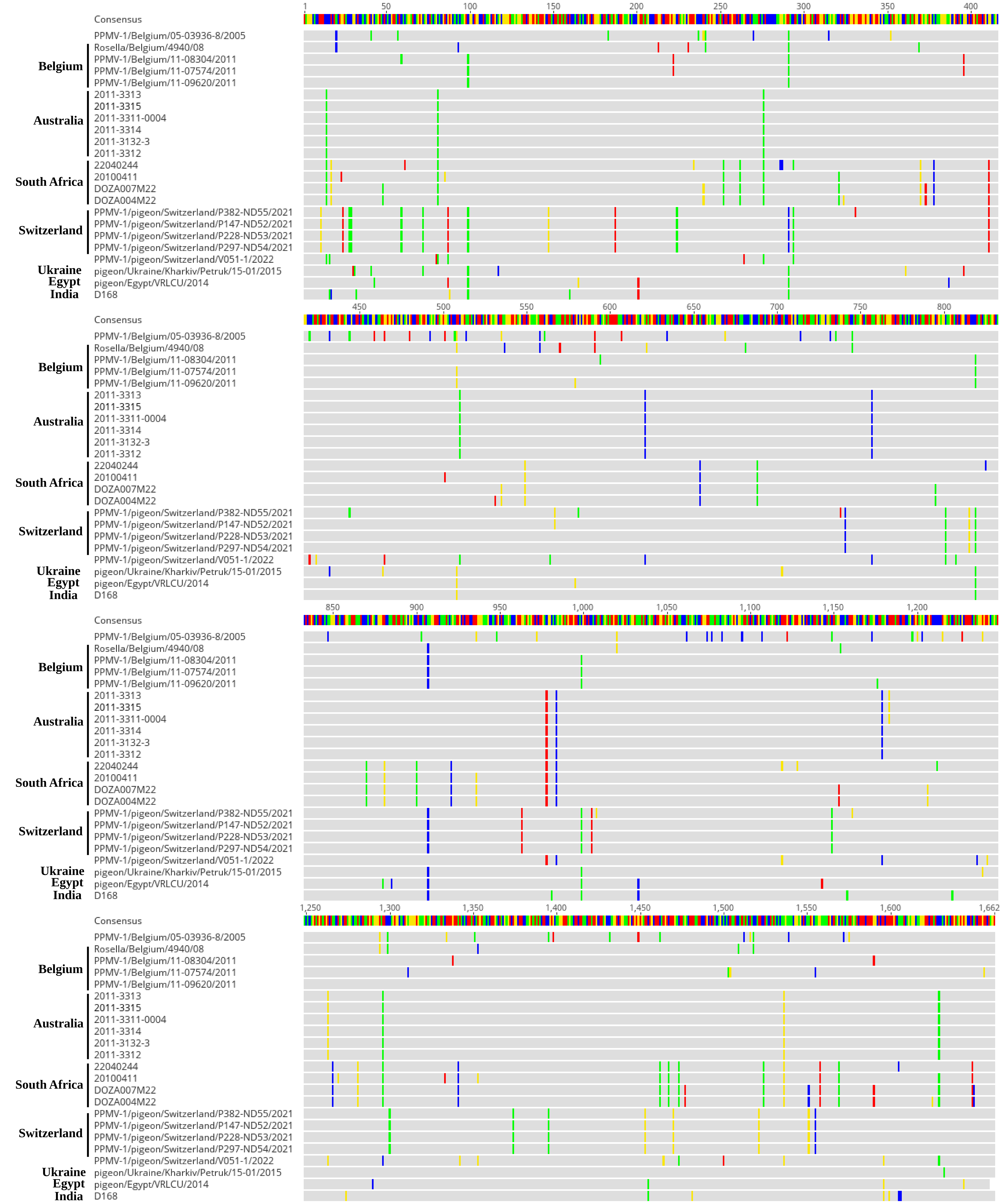

Comparison of nucleotide of F gene CDS among sub-genotype VI.2.1.1.2.2 NDVs isolated from all countries except China. The first isolate, PPMV-1/Belgium/05-03936-8/2005, was set as reference. The 23 VI.2.1.1.2.2 NDVs isolated from all countries except China were contained. The red, blue, yellow, and green vertical line represented A, C, G, and T, respectively.
