# Supplementary material for: Global evolution dynamics of genotype VI NDVs and dissection of the biological properties of strains from the prevalent sub-genotypes
Source: J Virol. 2025 Dec 30;100(2):e01799-25. doi: 10.1128/jvi.01799-25 (PMC12911885; doi:10.1128/jvi.01799-25)
Supplement: Figure S3 — Comparison of nucleotide of F gene CDS among sub-genotype VI.2.1.1.2.2 NDVs isolated from China. [file jvi.01799-25-s0003.pdf]

Supplemental Figure 3

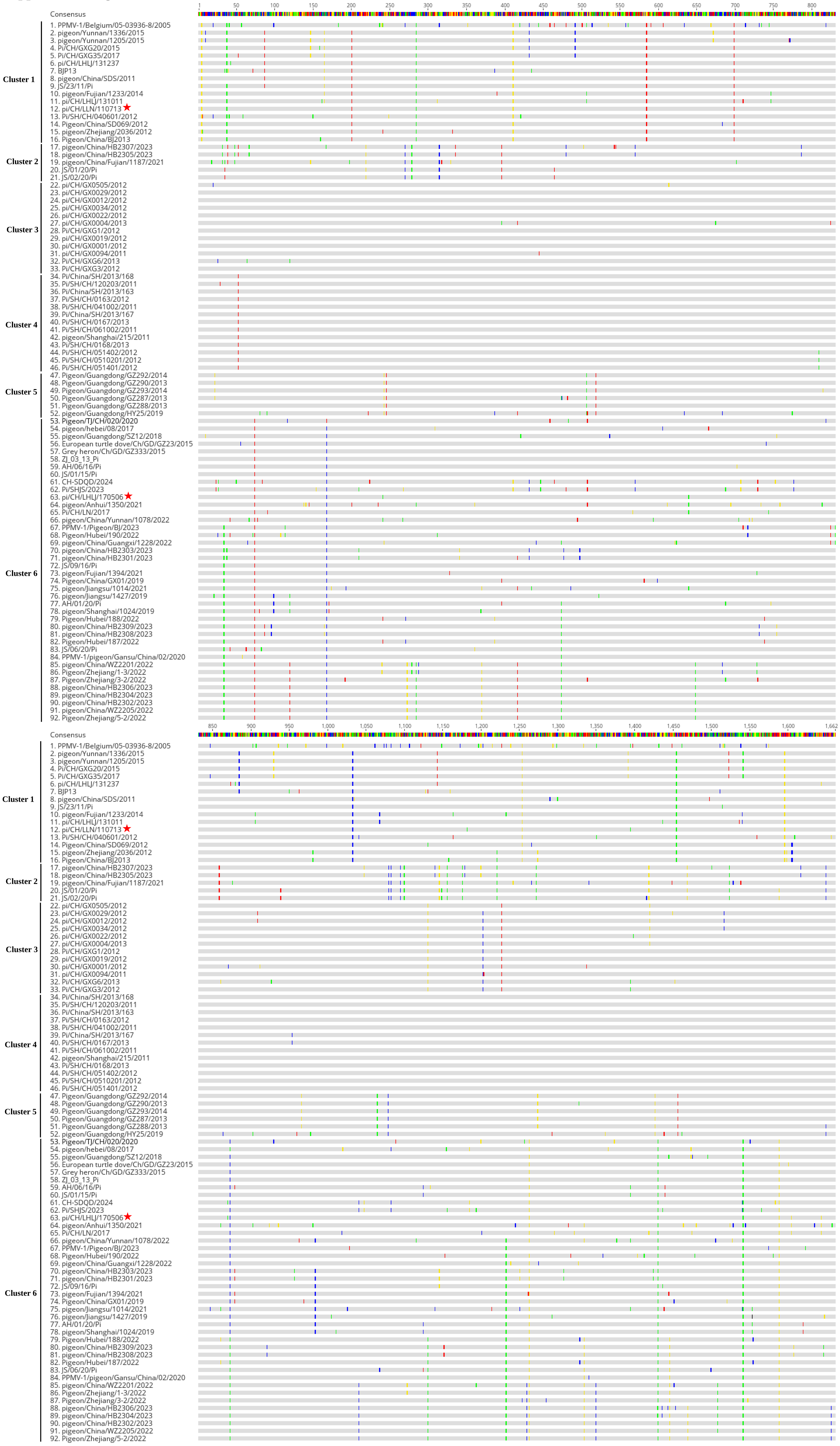

Comparison of nucleotide of F gene CDS among sub-genotype VI.2.1.1.2.2 NDVs isolated from China.
The first isolate, PPMV-1/Belgium/05-03936-8/2005, was set as reference. At least five different isolates which presented identical nucleotide substitution pattern were grouped into one cluster. The isolates without classification into the defined clusters were not shown. The red, blue, yellow, and green vertical line represented A, C, G, and T, respectively. The isolates used for subsequent research was labelled with red pentastars.
