# Supplementary material for: Global evolution dynamics of genotype VI NDVs and dissection of the biological properties of strains from the prevalent sub-genotypes
Source: J Virol. 2025 Dec 30;100(2):e01799-25. doi: 10.1128/jvi.01799-25 (PMC12911885; doi:10.1128/jvi.01799-25)
Supplement: Figure S4 — Comparison of deduced amino acid sequences of F protein among sub-genotype VI.2.1.1.2.2 NDVs isolated from China. [file jvi.01799-25-s0004.pdf]

Supplemental Figure 4

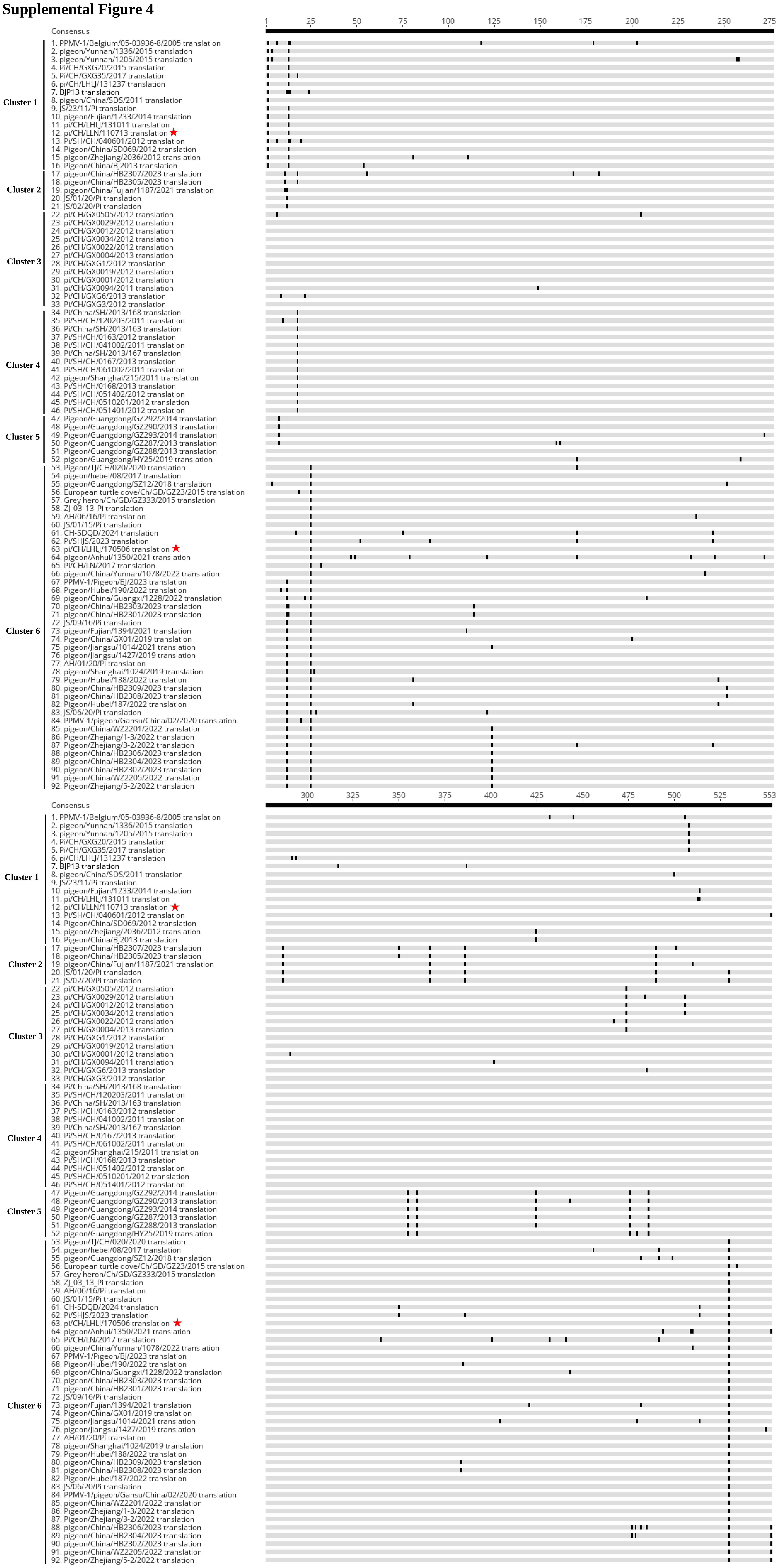

Comparison of deduced amino acid sequences of F protein among sub-genotype VI.2.1.1.2.2 NDVs isolated from China. The first isolate, PPMV-1/Belgium/05-03936-8/2005, was set as reference. The amino acids in each virus that were different from the consensus sequence among these viruses were indicated as black vertical line. The isolates used for subsequent research was labelled with red pentastar.
