# Supplementary material for: Global evolution dynamics of genotype VI NDVs and dissection of the biological properties of strains from the prevalent sub-genotypes
Source: J Virol. 2025 Dec 30;100(2):e01799-25. doi: 10.1128/jvi.01799-25 (PMC12911885; doi:10.1128/jvi.01799-25)
Supplement: Figure S1 — Phylogenetic analysis of genotype VI NDVs. [file jvi.01799-25-s0001.pdf]

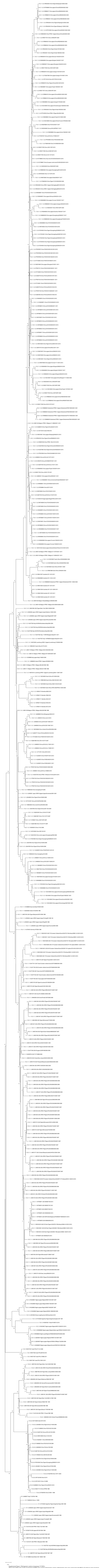

Supplemental Figure 1 Phylogenetic analysis of genotype VI NDVs.
The F gene CDSs of 412 strains of genotype VI NDV were used for phylogenetic analysis. Phylogenetic trees were constructed with MEGA 10 software using maximum likelihood with 1000 bootstraps.
